# Effects of maternal anthropometrics on pregnancy outcomes in South Asian women: a systematic review*

**DOI:** 10.1111/obr.12636

**Published:** 2018-01-19

**Authors:** E. Slack, J. Rankin, D. Jones, N. Heslehurst

**Affiliations:** ^1^ Institute of Health & Society Newcastle University Newcastle upon Tyne UK; ^2^ Health and Social Care Institute Teesside University Middlesbrough UK

**Keywords:** Ethnicity, gestational weight gain, maternal obesity, systematic review

## Abstract

**Aim:**

This systematic review investigates associations between maternal pre‐pregnancy/early‐pregnancy anthropometrics (e.g. weight and body fat), anthropometric change and pregnancy outcomes in South Asian and White women.

**Methods:**

Twelve electronic literature databases, reference lists and citations of all included studies were searched. Observational studies published in the English language were included. Descriptive synthesis was used to summarize the evidence base.

**Results:**

Twenty‐two studies met the inclusion criteria (403,609 births [351,856 White and 51,753 South Asian]). Nine were prospective cohort studies, nine were retrospective cohort studies and two were cross‐sectional studies. Results suggested that in South Asian women, maternal pre‐pregnancy/early‐pregnancy anthropometrics were associated with anthropometric change, birthweight, mode of delivery and gestational diabetes mellitus (GDM). Gestational anthropometric change was found to be associated with GDM. There was limited evidence to suggest that there may be associations between maternal pre‐anthropometrics/early anthropometrics and hypertensive disorders, stillbirth, congenital anomalies, post‐natal weight retention and post‐natal impaired glucose tolerance. The evidence suggested a combined effect of pre‐pregnancy/early‐pregnancy anthropometrics and gestational anthropometric change on both GDM and post‐natal weight retention.

**Conclusion:**

The increased risk of adverse pregnancy outcomes in South Asian women should be considered in guidelines for weight management before and during pregnancy.

**Abbreviations**
AOR: adjusted odds ratio
BMI: body mass index
CI: confidence interval
GDM: gestational diabetes mellitus
GWG: gestational weight gain
IGT: impaired glucose tolerance
IoM: Institute of Medicine
NICE: National Institute for Health and Care Excellence
NICU: neonatal intensive care unit
OR: odds ratio
PAF: population attributable fraction
PPH: post‐partum haemorrhage
SD: standard deviation
SFT: skin‐fold thickness
WHO: World Health Organization

## Introduction

Asian populations are at increased risk of obesity‐related comorbidities, e.g. diabetes and hypertension, at a lower body mass index (BMI) than are White populations 1. This is due, in part, to differences in body composition as BMI in some Asian populations reflects a higher body fat percentage than that in White populations does 2. The World Health Organization (WHO) BMI criteria reflect this difference in risk. For the general population, BMI criteria are underweight (<18.5 kg m^−2^), recommended weight (≥18.5 to <25 kg m^−2^), overweight (≥25 to <30 kg m^−2^) and obese (≥30 kg m^−2^) 3. The obese group can then be further subdivided into class I (≥30 to <35 kg m^−2^), class II (≥35 to <40 kg m^−2^) and class III (≥40 kg m^−2^) 3. However, the BMI criteria to indicate risk among Asian populations are reduced for recommended weight (≥18.5 to <23 kg m^−2^), overweight (≥23 to <27.5 kg m^−2^) and obese (≥27.5 kg m^−2^) 3. The obese group can be further subdivided into class I (≥27.5 to 32.5 kg m^−2^), class II (≥32.5 to <37.5 kg m^−2^) and class III (≥37.5 kg m^−2^) 2. It is possible that the pattern of higher obesity‐related risk may extend to pregnancy, resulting in increased risk of adverse pregnancy outcomes among Asian populations at a lower pre‐pregnancy/early‐pregnancy BMI and/or lower gestational weight gain (GWG).

Maternal obesity and both inadequate and excess GWG have been associated with adverse health outcomes for mother and infant 4. International research has highlighted that maternal obesity increases risks for both mother and child including gestational diabetes mellitus (GDM), pre‐eclampsia, gestational hypertension, depression, instrumental and caesarean birth, pre‐term and post‐term birth, large‐for‐gestational‐age babies, congenital anomalies and perinatal death 5, 6, 7, 8, 9. Maternal obesity has also been associated with longer‐term outcomes for the infant, such as subsequent obesity development and the associated life course morbidities 10. Excess GWG has also been associated with increased birthweight and foetal growth, caesarean delivery, childhood overweight and post‐natal weight retention 11, 12, 13, while inadequate GWG has been associated with decreased birthweight and foetal growth 11. The Institute of Medicine (IoM) in the USA have developed guidelines for GWG according to pre‐pregnancy/early‐pregnancy BMI category using the WHO general population BMI criteria 2. Recommended GWG ranges are 12.5–18 kg for women with an underweight BMI, 11.5–16 kg for recommended BMI, 7.5–11.5 kg for overweight and 5–9 kg for obese 14. The IoM guidelines are based on evidence of the weight gain‐related risk of caesarean delivery, birthweight, preterm birth, childhood obesity and post‐natal weight retention 14. This evidence is drawn from a variety of ethnic groups including White, African‐American, East Asian (including Chinese, Filipino and Japanese) and Hispanic populations.

Internationally, some countries such as Canada and Finland and regions of Australia have adopted the IoM guidelines 15. However, there are currently no UK guidelines for GWG. The National Institute for Health and Care Excellence (NICE) has not incorporated the IoM recommendations into national guidelines for weight management during pregnancy owing to a lack of UK population evidence, particularly relating to ethnic diversity 16. NICE recommended that research was needed to investigate weight gain in pregnancy and health outcomes among UK ethnic minority groups 16. In the UK, the largest ethnic group is White (86.0% of the population) followed by Asian (7.5% of the population) 17, 18. The majority of the UK Asian population are South Asian, Indian (2.5%), Pakistani (2.0%) and Bangladeshi (0.8%) 17, 18. NICE defines the UK South Asian population as ‘immigrants and descendants from Bangladesh, Bhutan, India, Indian‐Caribbean (immigrants of South Asian family origin), Maldives, Nepal, Pakistan and Sri Lanka’ 19. The populations used to develop the IoM guidelines do not include South Asian populations, and, therefore, the IoM guidelines may not be appropriate for ethnic minority groups in the UK. Additionally, current UK guidelines for both weight management 16 and the clinical management of obesity‐related risks in pregnancy 20 use the WHO general population BMI criteria rather than incorporating the BMI criteria specific to Asian populations. National data from England show that the incidence of maternal obesity in South Asian populations doubles when using ethnic group‐specific BMI criteria rather than general population BMI criteria 21. Therefore, a large proportion of South Asian women are potentially being incorrectly assigned to low‐risk care using current UK guidelines, which may widen the gap in health inequalities in access to health care 21.

Variations in obesity‐related risk by ethnicity lead to health inequalities 22; addressing these inequalities requires an accurate account of epidemiology 23. There are existing reviews that consider associations between maternal BMI and pregnancy outcomes 6, 24 as well as GWG and pregnancy outcomes 11, 14. However, there is a lack of systematically reviewed evidence that relates to South Asian women or considers additional measures of body composition (anthropometrics) such as skin‐fold thickness (SFT) and body fat percentage. These measures are especially important for Asian populations given the association between risk and body fat percentage and distribution. This systematic review aimed to synthesize the existing evidence base of associations between maternal pre‐pregnancy/early‐pregnancy anthropometrics (e.g. BMI and SFT. From this point forward exposures of pre‐pregnancy/early‐pregnancy BMI, SFT etc will be referred to as pre‐pregnancy) and/or gestational change in anthropometrics (e.g. GWG and gestational change in SFT) on pregnancy outcomes among South Asian women and their offspring compared with White women.

## Methods

Electronic databases were searched using keywords. Search terms and subject headings were converted into the relevant format for 12 databases: MEDLINE (Fig. S1), Embase, Scopus, PsycINFO, British Nursing Index, the Cumulative Index to Nursing and Allied Health Literature, Allied and Complementary Medicine Database, the Joanna Briggs Institute database, PROSPERO, Centre for Reviews and Dissemination database, Cochrane Database of Systematic Reviews and the federated search engine Epistemonikos, which provides access to systematic reviews, and primary studies included in these reviews (all searches shown in Fig. S1).

The search strategy for this review was designed to maximize the identification of relevant epidemiological studies. Additional searches included hand searching the reference lists of relevant studies or related reviews identified by the database searches to identify any relevant studies that had been cited. Each study that met the inclusion criteria was subjected to citation searches using all citations produced by Google Scholar to identify any published studies that had cited the included studies. Authors of any relevant published abstracts were contacted to identify any subsequent full publications of the research. Any studies identified by the supplementary searches were also subject to reference list and citation searches until no further eligible studies were identified. Authors of the final included studies were contacted for additional data to include in the analyses when required.

The comprehensive search strategy was carried out between December 2015 and July 2017. Inclusion criteria were peer‐reviewed full studies, published in the English language at any date. Studies involving observational quantitative research methods including cross‐sectional, case control and cohort study designs were included. Studies had to include data for both South Asian women and White women, and either

1. maternal pre‐pregnancy or early‐pregnancy anthropometric measures such as BMI or SFT, and pregnancy outcomes for mother or infant;
2. a measure of gestational anthropometric change (i.e. change from pre‐pregnancy measures at specific time points in the pregnancy) such as GWG or gestational change in SFT, and pregnancy outcomes for mother or infant.

South Asian women were defined as per 2013 NICE guidelines to represent UK South Asian populations 19. Studies were also included if they were carried out in the UK and referred to an Asian population, because in the UK the term Asian is used to refer to people with ancestry in the Indian subcontinent, whereas in other countries, the meaning is much broader, particularly in the USA where the term is mainly used to describe East Asian populations 25. White populations were defined as White, White European, Caucasian or White British women. In studies that reported UK data and more than one White or European ethnic group, the data for White British or UK participants were included in this systematic review.

Two authors (E. S. and D. J.) independently screened titles, abstracts and full papers of potentially relevant studies for inclusion in the review. All four authors then carried out data extraction and quality assessments for all included studies (E. S., D. J., J. R. and N. H.). A standardized protocol was used for data extraction (Fig. S2). Quality assessment utilized an adapted version of the Newcastle–Ottawa scale for cohort studies 26. Full details of the exact questions used and reasons for amendments to the scale are given in Fig. S3. The Newcastle–Ottawa quality assessment scale is a tool for assessing the quality of non‐randomized studies (where a score of 0 is lowest quality and a score of 8 it the highest possible quality) 26. It has previously been used for a systematic review considering the association between maternal BMI and pregnancy outcomes 7. Independent data extractions and quality assessments were combined and agreed upon. References were managed and recorded in endnote version 7 (1988–2016 Thompson Reuters, New York, USA).

Appropriateness of pooling the results of the individual studies identified for inclusion in the systematic review was assessed. Owing to the heterogeneity of pregnancy outcomes, anthropometric measures and comparisons made (i.e. South Asian women compared with White women of same BMI, and women of the same BMI compared within ethnic groups) in the primary studies, pooling of the data was not appropriate, and meta‐analysis was not possible. A descriptive synthesis has been used to provide a narrative summary of pregnancy outcomes by weight‐related exposure groups: group 1, maternal pre‐pregnancy anthropometrics; group 2, gestational change in anthropometrics during pregnancy; and group 3, a combination of maternal pre‐pregnancy anthropometrics and gestational change in anthropometrics. The systematic review was registered on the PROSPERO database (reference 42015024801).

## Results

**Figure 1:**
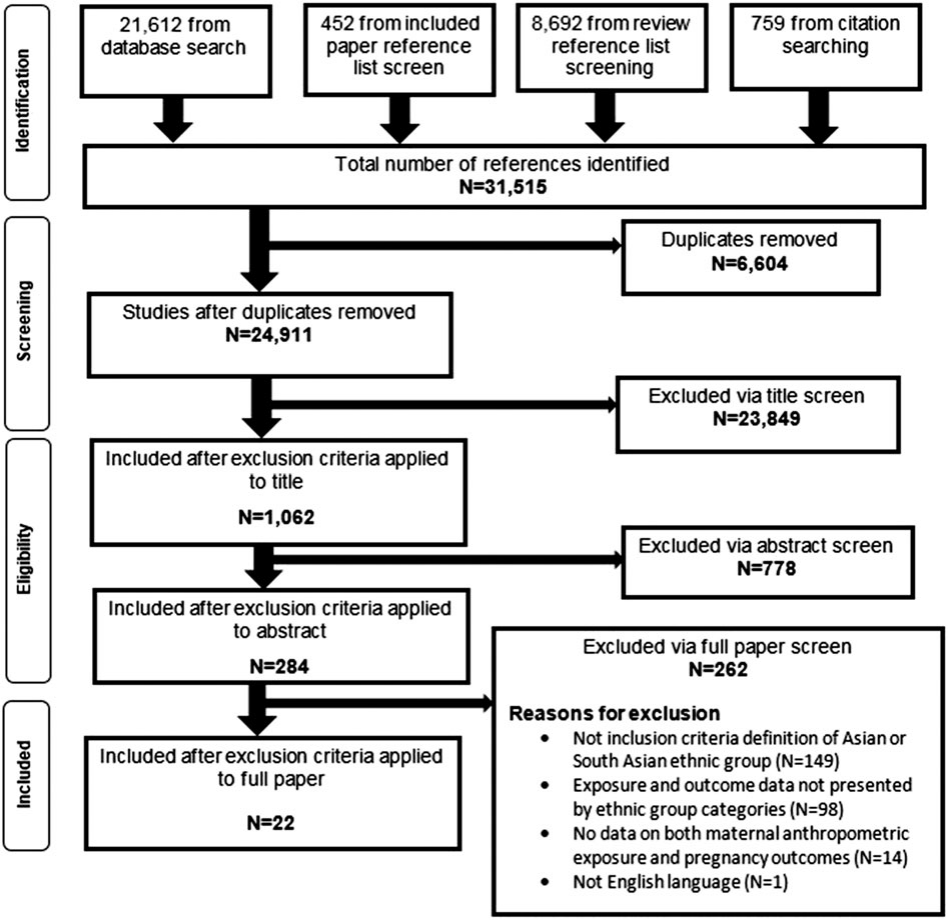
Preferred Reporting Items for Systematic Reviews and Meta‐Analyses flowchart of searches, screening, and inclusion and exclusion of studies.

A total of 31,515 studies were identified by the searches, of which 22 met the inclusion criteria (Fig. 1) and included a total of (403,609 births [351,856 White and 51,753 South Asian]). Thirteen studies were from the UK 27, 28, 29, 30, 31, 32, 33, 34, 35, 36, 37, 38, 39, three from Australia 40, 41, two from Norway 42, 43 and Canada 44, 45, and one from USA 46 and Spain 47 (Table 1). Some studies used more than one exposure: 21 studies used maternal anthropometric measurements as the exposure 27, 28, 29, 30, 31, 32, 33, 34, 35, 36, 37, 38, 39, 40, 41, 43, 44, 45, 46, 47, 48 (group 1), three used gestational change in maternal anthropometrics as the exposure 31, 42, 47 (group 2) and two used a combination of pre‐pregnancy anthropometrics and gestational change in anthropometrics (42, 43) (group 3). Of the studies that used maternal BMI as a categorical exposure (*n* = 7), only three studies applied BMI criteria for South Asian populations 29, 46, 48. The quality scores of the studies ranged from 2 to 7 (out of a maximum of 8), with a mean score of 5 (Table 1). Detailed quality assessment results are presented in Table S4. In addition, there was one study carried out in Australia that defined South Asian women as per the NICE guidelines but also included women from Afghanistan and Iran. As the majority of the South Asian population met the NICE definition, and owing to the ambiguity of definitions used by other studies (e.g. ‘from the Indian subcontinent’ or ‘any other Asian background’), a decision was made to include this study in the review.

**Table 1:** Summary of included studies [Colour table can be viewed at http://wileyonlinelibrary.com]

Author, publication year, Region and country, Study design	**Ethnic groups** (Ethnic group terms and definitions used by included article, and sample size, n)	Data collection time period	Exposure	Outcome	Quality score (out of 8)
Anand *et al* 2015, Ontario, Canada, Prospective cohort 45	White Caucasian, n=401 South Asian, n=389 Total n=790	White participants: 8 October 2002 and 8 July 2009. South Asian participants: 11 July 2011 and 20 September 2013	• Maternal weight (kg) and BMI (kg/m^2^)	GWG (kg)	5
Bissenden et al, 1981, Birmingham, UK, Prospective cohort 31	European n=28 Asian; Pakistani or Bangladeshi, n=11 Total n=39	Not specified	• Incremental changes per week in body measurements in the second trimester	Well grown babies	2
			• Maternal weight		
			• Mid upper arm circumference		
			• Triceps, biceps and subscapular skinfold thickness		
Bissenden *et al* 1981 Birmingham, UK, Prospective cohort 30	European, n=31 Asian; Pakistani or Bangladeshi, n=39 Total n=70	Not specified	• Maternal weight	Anthropometric change; Incremental changes per week in body measurements in the second trimester; Maternal weight; Mid upper arm circumference; Triceps, biceps and subscapular skinfold thickness	2
			• Triceps, biceps and subscapular skinfold thickness		
			• Incremental change from booking to 29 weeks was also calculated		
Bryant et al, 2014, Bradford, UK, Prospective cohort 28	White British n=4547 Pakistani n=4547 Total n=8478	March 2007 to December 2010	• Maternal BMI (Defined using WHO classification (BMI≥30kg/m2) and South Asian specific category (BMI ≥27.5kg/m^2^))	Mode of birth; Hypertensive disorders of pregnancy; GDM Macrosomia; Preterm births	5
Davies‐Tuck *et al* 2016 Australia, Retrospective cohort 48	White; Australian and New Zealand n=18768 South Asian; Bangladesh, Pakistan, Bhutan, India, Maldives, Nepal, Iran, Afghanistan, Sri Lanka n=8342 Total n=27110	2009 to 2013	• Maternal BMI (Obesity is BMI more than or equal to 30kg/m^2^ (also did analysis on Asian population using BMI cut off of 26kg/m^2^. However, this did not change the results and so only a BMI cut off of 30kg/m^2^ is presented))	Gestational hypertension; GDM; Preterm birth; Shoulder dystocia; Postpartum Haemorrhage (1000ml); Mode of delivery (induced labour, instrumental vaginal, unplanned caesarean section); Birthweight (small for gestational age (<10^th^ centile) and macrosomia (>4kg)); Fetal compromise (pregnancy or labour); Admission to NICU/SCN; Any perinatal morbidity; Stillbirth	7
Dornhorst *et al* 1992 London, UK, Prospective cohort 35	White; Northern European and Caucasian n=6109 Indian; from the Indian subcontinent n=1164 Total n=7273	1984 ‐1988	• Maternal BMI (kg/m^2^, <27 and more than 27)	GDM	5
Dunne *et al* 2000 Birmingham, UK, Retrospective cohort 38	Caucasian n=312 Indo‐Asian women; Pakistan, India, Bangladesh, n=128 Total n=440	1990‐1998	• Maternal BMI (kg/m^2^)	GDM and impaired glucose tolerance	3
Hernandez‐Rivas *et al* 2013 Barcelona, Spain, Prospective cohort 47	Caucasian n=190 South Central Asian; Pakistan, India, Bangladesh n=81 Total n=271	January 2004 to April 2011	• Maternal BMI (kg/m^2^)	GDM	4
			• Weight gain during pregnancy (kg)		
Nishikawa *et al* 2017 London, UK, Retrospective cohort 39	White; British, English, European, White‐Other, n=26433 South Asian; Asian, British‐Asian, Asian‐Other, Indian, Bangladeshi, Pakistani, Sri Lankan, n=2957 Total n=29390	2004‐2012	• Maternal BMI (kg/m^2^)	Presence or absence of diabetes during pregnancy	7
Makgoba *et al* 2011, London, UK, Retrospective cohort 33	White woman, n=131201 South Asian women, n=2749 Total n=134150	1988 and 2000	• Maternal BMI (kg/m^2^)	GDM	5
Makgoba *et al* 2012 London, UK, Retrospective cohort 34	White woman, n=107901 South Asian women, n=15817 Total n=123718	1988 and 2000	• Maternal BMI (kg/m^2^)	GDM; Birthweight	5
Oteng‐Ntim *et al* 2013 London, UK, Cross sectional 32	White; White British, White Irish and Other White, n=12418 Asian; Bangladeshi, Indian, Pakistani, other Asian and Asian British, n=1162 Total n=13580	Jan 1^st^ 2004 and Dec 31^st^ 2008	• Maternal BMI (kg/m^2^)	GDM; Mode of delivery; Postpartum haemorrhage; Preterm delivery; Macrosomia; Low birthweight; Admission to NICU/SCN; Perinatal death	7
Penn *et al* 2014 London, UK, Retrospective cohort 29	White; British, Irish, White Other, n=26390 Asian; Indian, Pakistani, Bangladeshi, Asian Other, n=2857 Total n=29347	January 2004 and May 2012	• Maternal BMI (kg/m^2^)	Stillbirth	6
			• Also created a second BMI variable for South Asian women only.		
Pu *et al* 2015 Northern California, Retrospective cohort 46	White; Non‐Hispanic White, n=9011 Asian Indian, n=5069 Total n=14080	Between 2007 and 2012	• Maternal BMI (kg/m^2^) (Also WHO categories relevant to South Asian women)	GDM	7
Retnakaran *et al* 2006 Canada, Cross sectional 44	Caucasian n=116 South Asian; India, Pakistan, Sri Lanka and Bangladesh, n=31 Total n=147	Not specified	• Maternal BMI (kg/m^2^)	GDM; Impaired glucose tolerance; Normal glucose tolerance	3
			• Weight gain in pregnancy (kg)		
			• Adiponectin concentration (measure of hypoadiponectinemia)		
Sharma *et al* 2011 Oxford, UK, Prospective cohort 36	White; British, Irish and any other White Background, n=709 Asian or Asian British; Indian, Pakistani, Bangladeshi or any other Asian background, n=249 Total n=958	February 2009 to December 2009	• Maternal BMI (kg/m^2^)	Distance from Skin to lumbar epidural space	4
Sheridan *et al* 2013 Bradford, UK, Prospective cohort 27	White British n=4488 Pakistani n=5127 Total n=9615	2007 and 2011	• Maternal BMI (kg/m^2^)	Congenital anomalies	5
Sinha *et al* 2002 Birmingham, UK, Retrospective cohort 37	Caucasian n=91 Indo Asian; Predominantly Muslim women from the Punjab Region, n=89 Total n=180	Not specified	• Booking weight (kg) (Booking defined as 16 weeks gestation)	GDM; Postpartum impaired glucose tolerance	4
Sommer *et al* 2015 Groruddalen, Oslo, Norway, Prospective cohort 43	European; Europeans of whom 82% were Norwegian (Three women born in North America were categorised as Europeans) n=353 South Asian; 63% Pakistani and 31% Sri Lankan n=190 Total n=543	May 2008 to May 2010	• Maternal BMI (kg/m^2^)	GDM; Anthropometric change during pregnancy; PPWR	5
			• Subcutaneous fat (mm, at 14 and 28 weeks gestation, and 14 weeks after delivery)		
			• Serum Leptin level (ug/l at 14 and 28 weeks gestation, and 14 weeks after delivery)		
Sommer *et al* 2014 Groruddalen, Oslo, Norway, Prospective cohort 42	European n=348 South Asian n=181 Total n=529	May 2008 and May 2010	• Maternal BMI (kg/m^2^)	GDM	6
			• Body weight (kg) and truncal fat		
			• Subcutaneous fat		
			• Weight gain, and gain of total fat, truncal fat and mean skinfold gain		
Wong *et al* 2011 New South Wales, Australia, Retrospective cohort 40	Anglo‐European n=215 South Asian; Indian, Pakistani, Sri Lankan and Fiji Indian n=160 Total n=375	July 2007 to July 2010	• Maternal BMI (kg/m^2^)	GDM	4
Yue *et al* 1996 Sydney, Australia, Retrospective cohort 41	Anglo‐Celtic n=2412 Indian n=114 Total n=2526	Not specified	• Maternal BMI (kg/m^2^)	GDM	4

Abbreviations: WHO=World Health Organization, BMI=Body mass index, GDM=Gestational diabetes mellitus, PPWR=Postpartum weight retention, NICU=Neonatal intensive care unit, SCN=Special care nursery

Overall, results were inconsistent. Results have been presented narratively and grouped by pregnancy outcome and type of exposure. Included studies reported 17 pregnancy outcomes: three during pregnancy (GDM, hypertensive disorders and gestational change in anthropometrics 1Gestational change in anthropometrics is an exposure to investigate associations with pregnancy outcomes and also an identified pregnancy outcome associated with pre‐pregnancy anthropometrics.); 12 perinatal outcomes (mode of delivery, distance from skin to epidural space, post‐partum haemorrhage [PPH], shoulder dystocia, foetal compromise, congenital anomaly, preterm birth, stillbirth, admission to the neonatal intensive care unit [NICU] or special care nursery, perinatal death, birthweight and a composite category for any perinatal morbidity); and two post‐natal outcomes (weight retention and impaired glucose tolerance IGT). Six of the outcomes were reported by more than three studies (GDM, anthropometric change during pregnancy, preterm birth, birthweight, mode of delivery and post‐partum weight retention), and four of the outcomes were reported by two studies (stillbirth, hypertensive disorders, admission to NICU/special care nursery and PPH). The remaining eight outcomes were reported by one study only (distance from skin to epidural space, post‐natal IGT, perinatal death, dystocia, foetal compromise, any perinatal morbidity, congenital anomalies and post‐natal weight retention).

### Gestational diabetes

#### Exposure group 1: maternal pre‐pregnancy anthropometrics

Thirteen studies reported data for maternal pre‐pregnancy weight, BMI, SFT and serum leptin levels 28, 32, 34, 35, 37, 38, 39, 40, 43, 44, 46, 47, 48. Nine reported an increased association with GDM for South Asian women compared with White women 28, 32, 34, 37, 38, 39, 40, 43, 47. Three found a decreased association 35, 44, 46. One found an increased association for South Asian women in unadjusted results 48. However, following adjustment, this association decreased to less than that for White women 48.

Six studies found that mean weight 37 or mean BMI 34, 38, 40, 43, 47 was lower in South Asian women with GDM compared with White women with GDM. Two studies reported statistical significance: one found the difference to be significant 34 and the other did not 47. One study reported that although South Asian women with GDM had a lower mean BMI than did White women with GDM, they had higher mean values of SFT and serum leptin levels 43 (Table 2).

**Table 2:** Pre‐pregnancy anthropometric measurements of women in population of women with GDM

Author and study year	Ethnic group	Exposure	Exposure mean (standard deviation)		*p* value
			White ethnic group	South Asian ethnic group	
Dunne *et al*., 2000 38	Caucasian women (*n* = 312) Indo‐Asian women: Pakistani, Indian, Bangladeshi (*n* = 128)	BMI (kg m^−2^)	29.2 (8.5)	29.1 (5.7)	—
Hernandez‐Rivas *et al*., 2013 47	Caucasian (*n* = 190) South Central Asian: Pakistani, Indian, Bangladeshi (*n* = 81)	BMI (kg m^−2^)	27.4 (6.18)	27.0 (4.65)	0.630
Makgoba *et al*., 2012 34	White European (*n* = 707) South Asian (*n* = 304)	BMI (kg m^−2^)	26.7 (5.8)	25.3 (4.9)	<0.001
Wong *et al*., 2011 40	Anglo‐European women (*n* = 215) South Asian women: Indian, Pakistani, Sri Lankan, Fiji Indian (*n* = 160)	BMI (kg m^−2^)	30.6 (8.1)	26.8 (5.2)	—
Sinha *et al*., 2003 37	Caucasian women (*n* = 91) Indo‐Asian women: predominantly Muslim women from the Punjab Region (*n* = 89)	Weight (kg)	69.8 (4.2)	68.3 (6.45)	—
Sommer *et al*., 2015 43	European (*n* = 353) South Asian: Pakistani and Sri Lankan (*n* = 543)	BMI (kg m^−2^)	27.3 (95% CI 25.9, 28.6)	25.5 (95% CI 24.3, 26.6)	—
		Sum of SFT (mm)	76.1 (95% CI 71.1, 81.0)	78.0 (95% CI 73.3, 82.7)	—
		Serum leptin (ng)	1.73 (95% CI 1.45, 2.04)	1.94 (95% CI 1.70, 2.20)	—

BMI, body mass index; CI, confidence interval; GDM, gestational diabetes; SFT, skin‐fold thickness.

Six studies presented odds ratios (ORs) or relative risks for the association between maternal pre‐pregnancy anthropometrics and GDM; three found an increased association between GDM and maternal BMI in South Asian women compared with White women 28, 32, 34, and two found a decreased association for South Asian women 35, 46. Two studies only presented unadjusted results; both of these studies found that ORs of GDM were higher in South Asian women than in White women 28, 34. Two studies presented unadjusted and adjusted results. One found that ORs of GDM were lower for South Asian women than were those for White women 35. The other found that South Asian women had a higher OR of GDM; however, following adjustment, the adjusted OR (AOR) decreased to less than that of White women 48. Two studies only presented adjusted results: one found the AOR was higher in South Asian women 32, and the other found that South Asian women had a lower AOR of GDM than did White women at the same BMI 46 (Table 3).

**Table 3:** Effects of maternal BMI on pregnancy outcomes in South Asian and White women

					OR (95%CI)		AOR (95%CI)	
Pregnancy outcome		Author and study year	Exposure	Control group	White ethnic group+	South Asian ethnic group+	White ethnic group+	South Asian ethnic group+
**GDM**		Bryant *et al* 2014 28	5kg/m^2^ increase in BMI	n/a	1.25 (1.12, 1.40)*	1.55 (1.43, 1.69)*	‐	‐
		Davies‐Tuck *et al* 2016 48	≥30kg/m^2^	<30kg/m^2^	3.20 (2.80, 3.65) ∞, *	3.48 (2.88, 4.21) ∞, *	3.21 (2.80, 3.67)*, 1	1.89 (1.57, 2.28)*, 1
		Dornhorst *et al* 1992 35	BMI ≥27 kg/m^2^	BMI <27 kg/m^2^	4.6 (2.1,10.4)*	3.5 (2.0, 4.2)*	4.3 (1.9, 9.8)*, 2	2.0 (0.9, 4.2)^2^
		Makgoba *et al* 2011 33	25.0‐29.9 kg/m^2^	15.5‐24.9kg/m^2^	1.77 (1.50, 2.09)*	2.57 (2.02, 3.23) ∞, *	‐	‐
			≥30kg/m^2^		4.70 (3.98, 5.55)*	5.80 (4.36, 7.71) ∞, *	‐	‐
		Oteng‐Ntim 2013 32, &	≥30kg/m^2^	<30kg/m^2^	‐	‐	4.97 (3.39, 7.28)*, 3 **PAF** 20.3 (15.46, 24.53)	5.48 (2.43, 12.35)*, 3 **PAF** 17.37 (13.07, 21.09)
		Pu *et al* 2015 46	≥25kg/^2^	<25kg/m^2^	‐	‐	2.0 (1.74, 2.4)*, $, 4 **PAF** 28.9 (22.4, 35.1)	1.17 (1.5, 2.0)*, $, 4 **PAF** 25.5 (17.4, 33.3)
			≥23kg/m^2^	<23kg/m^2^	‐	‐	‐	1.9 (1.7, 2.2)*, $, 4 **PAF** 39.0 (29.7, 47.6)
**Preterm delivery**		Bryant *et al* 2014 28	5kg/m^2^ increase in BMI	n/a	0.87 (0.77, 0.98)*	0.98 (0.87, 1.11)	‐	‐
		Davies‐Tuck *et al* 2016 48	≥30kg/m^2^	<30kg/m^2^	1.06 (0 .95, 1.18)∞	1.07 (0.80, 1.43)∞	1.04 (0.93, 1.16)^5^	1.08 (0.81, 1.45)^5^
		Oteng‐Ntim 2013 32	≥30kg/m^2^	<30kg/m^2^	‐	‐	1.66 (1.30, 2.11)*, 3 **PAF** 2.66 (1.06, 4.23)	1.25 (0.61, 2.56)^3^ **PAF** 2.39 (0.96, 3.81)
**Mode of delivery**	C‐section	Bryant *et al* 2014 28	5kg/m^2^ increase in BMI	n/a	1.34 (1.26, 1.42)*	1.36 (1.27, 1.45)*	‐	‐
	Unplanned C‐section	Davies‐Tuck *et al* 2016 48	≥30kg/m^2^	<30kg/m^2^	1.49 (1.37, 1.62)∞, *	1.37 (1.16, 1.62)∞, *	1.35 (1.23, 1.49)*, 6	1.38 (1.15, 1.66)*, 6
	Elective LSCS	Oteng‐Ntim 2013 32	≥30kg/m^2^	<30kg/m^2^	‐	‐	1.41 (1.08, 1.84)*, 3 **PAF** 4.24 (2.43, 6.00)	1.52 (0.73, 3.14)^3^ **PAF** 4.02 (2.31, 5.70)
	Emergency LSCS		≥30kg/m^2^	<30kg/m^2^	‐	‐	1.98 (1.69, 2.33)*, 3 **PAF** 3.48 (2.65, 4.30)	0.65 (0.32, 1.31)^3^ **PAF** 2.93 (2.23, 3.63)
	Induced labour	Davies‐Tuck *et al* 2016 48	≥30kg/m^2^	<30kg/m^2^	1.48 (1.37, 1.58)∞, *	1.29 (1.10, 1.50)∞, *	‐	‐
	Instrumental delivery		≥30kg/m^2^	<30kg/m^2^	0.65 (0.59, 0.72)∞, *	0.60 (0.48, 0.74)∞, *	0.73 (0.65, 0.82)*, 7	0.76 (0.57, 1.01)^7^
	Instrumental Delivery	Oteng‐Ntim 2013 32	≥30kg/m^2^	<30kg/m^2^	‐	‐	0.78 (0.63, 0.96)*, 3 **PAF** ‐1.84 (‐2.71, ‐0.98)	1.04 (0.50, 2.16)^3^ **PAF** ‐1.57 (‐2.30, ‐0.84)
**Birthweight**	Macrosomia	Bryant *et al* 2014 28	5kg/m^2^ increase in BMI	n/a	1.36 (1.27, 1.47)*	1.57 (1.41, 1.75)*	‐	‐
		Davies‐Tuck *et al* 2016 48	≥30kg/m^2^	<30kg/m^2^	1.43 (1.31, 1.56)∞, *	2.22 (1.78, 2.77) ∞, *	1.90 (1.73, 2.08)*, 8	2.24 (1.75, 2.83*, 8
		Oteng‐Ntim 2013 32	≥30kg/m^2^	<30kg/m^2^	‐	‐	1.54 (1.27, 1.89)*, 3 **PAF** 5.15 (3.64, 6.64)	0.98 (0.30, 3.20) **PAF** 5.52 (3.84, 7.18)
	SGA	Davies‐Tuck *et al* 2016 48	≥30kg/m^2^	<30kg/m^2^	0.64 (0.57, 0.72)∞, *	(0.56 (0.45, 0.71)∞, *	0.64 (0.57, 0.72)*, 9	0.64 (0.51, 0.81)*, 9
	LBW	Oteng‐Ntim 2013 32	≥30kg/m^2^	<30kg/m^2^	‐	‐	0.75 (0.58, 0.98)*, 3 **PAF** ‐0.01 (‐0.10, 0.08)	0.92 (0.47, 1.37)^3^ **PAF** ‐0.03 (‐0.20, 0.14)

OR=odds ratio AOR= adjusted odds ratio, GDM=gestational diabetes, SGA=small for gestational age, LSCS=lower section caesarean section, LBW=low birthweight

+ For exact ethnic group definitions please refer to Table 1

& GDM for this study includes GDM and pre‐existing diabetes

∞ Effect size calculated from data provided in published paper using STATA 14

* Significant as 95% confidence interval does not cross 1.00

$ Relative risk

PAF: population attributable fraction % and 95%CI (PAF is the reduction in population disease risk or mortality that would occur if the exposure to a risk factor was eliminated or reduced to an ideal exposure scenario, where the distributions of other risk factors in the population remain unchanged 50, 51)

1 adjusted for age, parity and smoking status

2 adjusted for age and parity

3 adjusted for age parity and deprivation

4 adjusting for maternal education, parity, smoking and insurance status

5 adjusted for age, parity and smoking

6 adjusted for maternal age, parity, account class, previous caesarean, onset of labour, gestation, birthweight, augmentation, epidural

7 adjusted for maternal age, parity, onset of birth, epidural, baby birthweight, gestation, head position, augmentation and account class

8 adjusted for parity, maternal age, account class, smoking, gestation and baby gender

9 adjusted for maternal age, parity, smoking and account class

One study found that South Asian women had lower insulin sensitivity relating to increasing BMI compared with White women (slope of −0.17 [95% confidence interval {CI} −0.22 to −0.13] in White, slope of −0.04 [95% CI −0.15 to 0.08] in South Asians) 44.One study presented the performance parameters (sensitivity, specificity, positive predictive value and negative predictive value) of different BMI cut‐offs (20, 21.5, 23, 25, 27.5 and 30 kg m^−2^) for White and South Asian women 39. These parameters gave an indication of the proportion of the population with GDM in each ethnic group that was captured using each cut‐off. A BMI of 20 kg m^−2^ captured 95.2% of White women compared with 95.0% of South Asian women with GDM 39. All other BMI cut‐offs investigated captured a higher percentage of South Asian women with GDM than White women 39. This ranged from 93.6% in South Asian women compared with 87.5% in White women at BMI 21.5 kg m^−2^, and 34.7% in South Asian women compared with 32.1% in White women at BMI 30 kg m^−2^ 39. The prevalence of diabetes in pregnancy was also estimated for each BMI cut‐off; results showed an increased risk of diabetes at all BMI points for South Asian women compared with White women 39. In addition, this study found that for South Asian women, the BMI with risk of GDM equivalent to 30 kg m^−2^ in White women was approximately 21 kg m^−2^ 39.

#### Exposure group 2: gestational anthropometric change

Two studies presented results for the association between GDM and gestational change in weight, fat mass, truncal fat and mean skin‐fold 42, 47. Both studies found an increased association with GDM in South Asian women compared with White women 42, 47. One identified a lower mean GWG among South Asian women with GDM (mean 8.3 kg, standard deviation [SD] 4.2) compared with White women with GDM (mean 9.4 kg, SD 4.96), although this was not significant (*p* = 0.163) 47. One study found that, compared with White women, South Asian women had significantly increased association with GDM and GWG, fat mass gain, truncal fat gain and mean skin‐fold gain, which remained significant following adjustment 43 (Table S5).

#### Exposure group 3: a combination of pre‐pregnancy anthropometrics and gestational change in anthropometrics

One study reported the combined influence of maternal pre‐pregnancy BMI and gestational gain in truncal fat on GDM 42. South Asian women had an increased OR of GDM (OR 2.86, 95% CI 1.88, 4.34) compared with White women. Within the ethnic groups, the ORs of GDM increased with a 1 SD increase in pre‐pregnancy BMI for both South Asian women (OR 4.75, 95% CI 2.96, 7.60) and White women (OR 1.66, 95% CI 1.4, 1.97). A similar pattern was observed for a 1 SD increase of truncal fat gain for South Asian (OR 3.8, 95% CI 2.4, 6.0) and White women (OR 1.3, 95% CI 1.1, 1.6), an increase in both truncal fat gain and pre‐pregnancy BMI among South Asian (OR 6.3, 95% CI 3.74, 10.63) and White women (OR 2.21, 95% CI 1.68, 2.89) 42. For all levels of exposure, the ORs of GDM were higher for South Asian women than for White women 42.

### Anthropometric change during pregnancy (outcome)

#### Exposure: maternal pre‐pregnancy anthropometrics

Three studies provided results for the association between the exposure of pre‐pregnancy BMI, weight, SFT and serum leptin levels and the outcome of gestational anthropometric change 30, 43, 45. One study presented GWG by pre‐pregnancy weight 45. One study presented mean difference in gain of maternal weight and SFT (bicep, triceps and subscapular) and did not present data on statistical significance 30. One study presented the change in BMI, triceps, subscapular, suprailiac SFT measures and the sum of all these, and also serum leptin levels from 14 to 28 weeks' gestation 43.

All three studies 30, 43, 45 identified that South Asian women had a lower pre‐pregnancy weight (or BMI) compared with White women (*p* = 0.015 43, *p* ≤ 0.0001 45). However, gestational change in weight or BMI was higher among South Asian women (*p* = 0.023 43, *p* = 0.17 45). Two studies presented some conflicting results relating to pre‐pregnancy baseline SFTs and gestational change in SFTs. One study found that measures of SFT (bicep, tricep and subscapular) at 8–18 weeks' gestation were higher for South Asian women compared with White 30. Another study also reported significantly higher subscapular SFT (*p* = 0.002) among South Asian women at 14 weeks, but no significant difference at 14 weeks for tricep (*p* = 0.83), suprailiac (*p* = 0.96) or the sum of SFTs (*p* = 0.20) 43. Results relating to change in SFT during pregnancy were also conflicting. One study reported that both bicep and tricep SFT gains were higher for South Asian women than for White women at all time points (29, 32 and 37 weeks) 30, while another study found no significant difference in tricep (*p* = 0.085) or suprailiac (*p* = 0.24) SFT at 28 weeks 43. However, similar results were present for subscapular SFT gain, which both studies reported to be higher at 28 weeks (*p* < 0.001) 43 and 29 weeks 30. One study further reported South Asian women had gained a significantly higher sum of triceps, subscapular and suprailiac SFTs (*p* = 0.001) 43. This study also found that serum leptin levels were significantly higher at 14 and 28 weeks' gestation in South Asian women (*p* = 0.002 and *p* ≤ 0.001 respectively) and that the change in serum leptin from 14 to 28 weeks' gestation was also significantly higher for South Asian women (*p* = 0.004) 43.

### Preterm birth

#### Exposure: maternal pre‐pregnancy anthropometrics

Three studies presented results for the association between pre‐pregnancy BMI and preterm birth (<37 weeks) 28, 32, 48. One reported unadjusted results, which suggested increased association between BMI and preterm birth among South Asian women (as the OR for preterm birth was significantly decreased for White women, but not for Pakistani women) 28. One reported adjusted results that suggested South Asian women have reduced OR of preterm birth with increased BMI compared with White women 32. One presented information for both unadjusted and adjusted results that suggested that with increased BMI, there was a marginally higher OR for preterm birth in South Asian women compared with White women 48. However, ORs (both unadjusted and adjusted) did not reach statistical significance for either ethnic group 48 (Table 3).

### Birthweight

#### Exposure: maternal pre‐pregnancy anthropometrics

Five studies reported results for the association between maternal pre‐pregnancy BMI, weight, mid upper arm circumference and SFT and birthweight 28, 31, 32, 34, 48. One study reported the outcome of well‐grown babies (above 10th centile 49) 31, three reported macrosomia 28, 32, 48, one low birthweight 32, one small for gestational age 48 and one birthweight *z* score 34.

South Asian women delivering well‐grown babies had significantly higher mean triceps and SFT (mm) than did White women (*p* < 0.025 and *p* < 0.005, respectively), but no difference in mean weight (kg), middle upper arm circumference (mm) and bicep SFT (mm) 31. Offspring of South Asian women had a higher AOR of low birthweight than did offspring of White women 32. White women with a BMI ≥ 30 kg m^−2^ also had significantly reduced AOR of low birthweight, while the reduction in AOR for South Asian women did not reach statistical significance 32. One study found that with increased BMI, the odds of small for gestational age decreased significantly for both South Asian and White women; there was minimal difference in the decrease between the two ethnic groups, and this remained the same following adjustment 48 (Table 3).

Two studies found that with increased BMI, South Asian women had a higher OR for macrosomia than did White British women 28, 48; for one study, this remained true following adjustment 48. However, the findings from one study refuted this association and reported South Asian women to have a lower AOR of macrosomia compared with White women (comparing maternal BMI ≥ 30 kg m^−2^ with BMI < 30 kg m^−2^) 32. One study found that in both women with and without GDM, BMI had a greater effect on birthweight *z* scores in South Asian women than in White women 34 (Table 3).

### Mode of delivery

#### Exposure: maternal pre‐pregnancy anthropometrics

Three studies reported results relating the association between maternal BMI and mode of delivery, including caesarean section and instrumental delivery 28, 32, 48. One found that South Asian women had a slightly higher OR of caesarean associated with an increase in BMI than did White women 28. One study found that the odds of unplanned caesarean were lower in South Asian women compared with White women 48; however, following adjustment, the odds in White women decreased below those of South Asian women 48. One study found that while there was a higher AOR of elective caesarean in South Asian women, the AOR of emergency caesarean was lower for South Asian women than for White women 32. Two studies presented results on instrumental delivery. Both studies found that South Asian women had a higher AOR of instrumental delivery associated with increased maternal BMI compared with White women 32, 48 (Table 3).

### Pregnancy outcomes with evidence available from one or two studies

Two studies reported data for each of the following outcomes: stillbirth, hypertensive disorders, admission to NICU/special care nursery and PPH. Single studies reported data for distance from skin to epidural space, post‐natal IGT, perinatal death, congenital anomalies and post‐natal weight retention. The results are presented in Table S5.

#### Exposure: maternal pre‐pregnancy anthropometrics

A significant positive association was identified between maternal BMI and stillbirth for South Asian infants but not for White infants in one study 29; the other study found a higher OR of stillbirth associated with maternal BMI for South Asian infants than for White (although this did not reach statistical significance) 48. Two studies found a significant positive association between maternal BMI and hypertensive disorders of pregnancy for both White and South Asian women, which was lower in South Asian women 28, 48; this association remained following adjustment in one of the studies 48. The ORs and AORs of admission of infants to NICU/special care nursery and maternal PPH were lower in South Asian women compared with White women 32, 48.

One study found that distance from skin to epidural space increased with increasing BMI; this was lower in South Asian women than in White women 36 (Table S5). In women with post‐natal IGT, mean weight was lower for South Asian women (68.3 kg) than for White women (79.9 kg) 37. There were higher AORs for offspring perinatal mortality associated with maternal BMI among South Asian infants than White infants 32. South Asian infants also had a higher OR of congenital anomalies at BMI 25–29.9 kg m^−2^ than did White infants, and a lower OR of congenital anomalies at BMI <18.5 and ≥30 kg m^−2^ (27). South Asian infants had higher ORs and AORs for shoulder dystocia and ‘any perinatal morbidity’ compared with White infants 48. ORs of foetal compromise were lower for South Asian infants; however, following adjustment, AORs for South Asian infants increased to more than those for White infants 48.

Post‐natal retention of anthropometric measures was found to be higher in South Asian women than in White women 43. At 14 weeks' gestation, South Asian women had significantly lower BMI (*p* = 0.015) and significantly higher subscapular SFT (*p* = 0.002) and serum leptin levels (*p* = 0.002) compared with White women 43. However, there was no significant difference in tricep SFT, suprailiac SFT or the sum of SFT (*p* = 0.83, *p* = 0.96 and *p* = 0.20, respectively) 43. Despite the differences at 14 weeks' gestation, South Asian women had significantly higher change in all measures: BMI (*p* ≤ 0.001), tricep SFT (*p* ≤ 0.001), subscapular SFT (*p* = 0.022), suprailiac SFT (*p* = 0.016), sum of SFT (*p* ≤ 0.001) and serum leptin levels (*p* ≤ 0.001) 43. At 14 weeks' gestation, BMI in South Asian women was not significantly different to that of White women (*p* = 0.83), and all other measures were significantly higher for South Asian women than for White women; South Asian women had significantly higher change in all measures: tricep SFT (*p* ≤ 0.001), subscapular SFT (*p* ≤ 0.001), suprailiac SFT (*p* = 0.001), sum of SFT (*p* ≤ 0.001) and serum leptin levels (*p* ≤ 0.001) 43.

## Discussion

This systematic review included 22 studies and data from 403,609 births to compare the association between pregnancy anthropometrics and pregnancy outcomes in South Asian and White women. The strongest evidence from the included studies suggests that South Asian women have a higher risk of GDM associated with maternal pre‐pregnancy anthropometrics and anthropometric change during pregnancy than do White women. There was also some evidence to suggest an increased association among South Asian women 2We are not comparing the results with White women here, just considering the association in South Asian women alone. with maternal pre‐pregnancy anthropometrics and the outcomes anthropometric change during pregnancy, birthweight, mode of delivery and GDM. Gestational anthropometric change was also found to be associated with GDM. We found limited evidence to suggest that there may be associations between maternal pre‐anthropometrics/early anthropometrics and hypertensive disorders, stillbirth, congenital anomalies, post‐natal weight retention and post‐natal IGT. The evidence also suggested that there may be a combined effect of pre‐pregnancy anthropometrics and gestational anthropometric change on both GDM and post‐natal weight retention. However, there was evidence that refuted some of these associations, and for many combinations of anthropometric exposure and pregnancy outcomes. Further evidence is required to explore these associations.

This review is the first to consider the association between maternal pre‐pregnancy anthropometrics and anthropometric change during pregnancy on pregnancy outcomes in migrant and descendant South Asian women. Included studies allowed exploration of three levels of anthropometric exposure (maternal pre‐pregnancy anthropometrics, gestational change in anthropometrics and a combination of maternal pre‐pregnancy anthropometrics and gestational change in anthropometrics) on a number of different pregnancy outcomes. Consideration of different anthropometric measures is particularly important in the South Asian population as they better reflect fat distribution than does BMI alone. In the non‐pregnant population, there is a wealth of evidence that the South Asian population is at an increased risk of diabetes at a lower BMI than the White population is 50.

It is hypothesized that these differences in body composition lower percentage of lean body mass and that a higher proportion of fat mass contributes to the increased risk of type 2 diabetes in the South Asian population 50. Included studies have identified that South Asian women have a higher risk of GDM at a lower BMI or weight than do White women 28, 32, 34, 37, 38, 40, 43, 47. A lower percentage of lean body mass and a higher proportion of fat mass may also play a role in the development of GDM at a lower BMI (or weight) than in White women. In addition to differences in body composition, many other factors may play a role in explaining the difference in risk of pregnancy outcomes observed between the White and South Asian populations, e.g. consanguinity 51, socioeconomic status 51, access to maternity care 52, maternal mental health 53, place of birth 51, maternal age 41 and marriage (or cohabiting status) 54.

A major strength of this review is the comprehensive search strategy used. We performed a search of 12 databases, piloting and refining the search strategy by the research team and an information scientist with expertise in database searching. We also searched citations and reference lists of included studies, and the reference lists of reviews that were related to the topic area. Of the 22 included studies, 20 were identified through database search alone 27, 28, 29, 30, 31, 32, 33, 34, 35, 36, 37, 38, 39, 40, 42, 43, 44, 45, 46, 48, one from screening the reference lists of relevant reviews 41 and one from searching the citations of included studies 47. In their recent review and meta‐analysis, Heslehurst *et al*. also found that in order to minimize bias, it was essential to include evidence from searches in addition to database searching 7.

The main limitation of this systematic review relates to data availability in the existing literature. Owing to the heterogeneity between populations (e.g. in relation to first‐trimester maternal obesity 21, blood pressure 55 and risk factors for coronary heart disease 56), we would have preferred to consider the risk for Pakistani, Indian and Bangladeshi women, etc. independently. However, the evidence identified did not allow for independent subgroup analysis, and all South Asian women had to be analysed together. This review was also unable to distinguish South Asian populations by place or birth and/or immigrant status. This has been shown to affect rates of GDM, as Asian and other immigrants to high‐income countries typically have higher rates of GDM than do those born in high‐income countries 57. Limited evidence was available for the following exposures: gestational anthropometric change and the combined effect of pre‐pregnancy anthropometrics and gestational anthropometric change; for measures of anthropometrics other than BMI and weight (e.g. SFT and body fat percentage) and for a number of pregnancy outcomes, evidence was only available from one or two studies. Although this was useful as it highlights that this is an under‐researched area, the lack of evidence available limited the ability for evidence synthesis and limits the conclusions that we can draw about these pregnancy outcomes.

Among the included studies that compared maternal BMI categories (*n* = 7), only three studies applied lower BMI cut‐offs specific to Asian populations 29, 46, 48. Consequently, the results from the studies using general population BMI criteria for the South Asian population may have underestimated the effect size and the significance of the association. This is due to women of potentially increased obesity risk (pre‐pregnancy BMI, 27.5‐30 kg m^−2^) not being included in the obese category and women of potentially increased overweight risk (BMI, 23–25 kg m^−2^) included in the ‘low‐risk’ comparison groups. This review has identified that South Asian women may have a higher risk of multiple‐pregnancy outcomes at the same BMI than do White women. Had the BMI categories specific to the South Asian population been applied, it is possible that this risk may be even higher, and that some pregnancy outcomes that this review found to be non‐significant for South Asian women may actually be higher and even reach statistical significance.

Of the three studies that did apply BMI cut‐offs for the South Asian population, one reported that using the Asian‐specific criteria did not make a difference to results, although it did not present the exact values 48. The other two studies both found a change in odds compared with the higher BMI cut‐off. Penn *et al*. found that the odds of stillbirth decreased from 4.64 (1.84, 11.70) for BMI ≥ 30 kg m^−2^ to 2.83 (1.17, 6.85) for BMI ≥ 27.5 kg m^−2^ 29. Despite the decrease in odds, South Asian women still had higher odds of stillbirth than do White women: 1.32 (0.68, 2.57) 29. Pu *et al*. found that the odds of GDM increased from 1.17 (1.5, 2.0) at BMI ≥ 25 kg m^−2^ to 1.9 (1.7, 2.2) at BMI ≥ 23 kg m^−2^ 46. Of all the studies that considered GDM as an outcome where maternal BMI was categorized as the exposure, Pu *et al*. found the lowest odds of GDM (for both BMI cut‐offs, Asian specific and general population) 46. One reason for this finding may be that women with overweight and obesity were grouped together in this study rather than examining the groups separately, or just looking at women with obesity.

This review has highlighted the lack of evidence available for the association between maternal pre‐pregnancy anthropometrics, gestational anthropometric change and pregnancy outcomes in South Asian women. More research is needed that explores both the individual and combined effects of maternal pre‐pregnancy anthropometrics and gestational anthropometric change on different pregnancy outcomes in South Asian women. No literature identified by this review considered the associations between maternal anthropometric exposures and childhood obesity among offspring. Childhood obesity was found to be an outcome associated with GWG in the 2009 IoM guidelines where the ethnic groups considered were White, African‐American, East Asian and Hispanic populations 14. Future research should investigate the association between maternal anthropometrics, both pre‐pregnancy/early‐pregnancy and the change during pregnancy, and childhood anthropometrics separately for boys and girls in the different South Asian subgroups. Future research investigating the association between maternal anthropometrics and pregnancy outcomes in South Asian women should present results separately for the different South Asian subgroups. Where possible, the risk associated with different anthropometric measures (as opposed to BMI alone) should be considered to inform the understanding of potential mechanisms relating to ethnic differences in body composition.

In addition, in this review, none of the included studies considered obesity subgroups using the Asian‐specific BMI criteria (27.5 to <32.5, ≥32.5 to <37.5, and ≥37.5 kg m^−2^ 2). It has been documented that within the pregnant population with obesity, levels of risk are not the same at all BMIs ≥ 30 kg m^−2^. For example, the risk of post‐term birth in class I obesity is different to that in class III obesity 7. In order to explore whether this is the same for the pregnant South Asian population, future research should investigate the risk of pregnancy outcomes within each of the obesity subgroups of the Asian‐specific BMI criteria, or ensure that BMI is investigated as a continuous exposure. It is recommended that wherever possible, BMI cut‐offs are used that would facilitate international comparisons (18.5, 20, 23, 25, 27.5, 30, 32.5, 35, 37.5 and 40 kg m^−2^) 2.

This review has highlighted that there are differences in obesity‐related risk in pregnancy for South Asian and White women. Results suggest that the risk of GDM is higher in South Asian women at a lower BMI than in White women, therefore supporting the use of ethnicity as independent criteria for GDM criteria in the NICE guidelines. Results also suggest that there are ethnic differences in risk for different pregnancy outcomes related to anthropometric measures. However, the lack of evidence available to explore these associations fully means that firm conclusions cannot be drawn. Therefore, before any recommendations can be made for policy and practice, more research is needed. In particular, the association between maternal anthropometrics and childhood obesity should be investigated in ethnic groups relevant to the UK population.

## Conclusion

This review found inconsistent results. However, evidence suggested that in South Asian women, maternal pre‐pregnancy anthropometrics are associated with anthropometric change during pregnancy, birthweight, mode of delivery and GDM. Evidence also highlighted that gestational anthropometric change may be associated with GDM. However, the limited evidence available for these exposures and also other pregnancy outcomes warrants further investigation to inform policy and practice to address health inequalities.

## Conflict of interest statement

No conflict of interest was declared.

## Supporting information


**Figure S1.** Search strategies
**Figure S2.** Data extraction form
**Figure S3.** Quality assessment
**Data S4** Quality assessment scores
**Data S5.** Effects of specified exposure on pregnancy outcomes in South Asian and White women
