# Supplementary material for: Effects of maternal anthropometrics on pregnancy outcomes in South Asian women: a systematic review
Source: Obes Rev. 2018 Jan 19;19(4):485–500. doi: 10.1111/obr.12636 (PMC5969310; doi:10.1111/obr.12636)
Supplement: Supplementary file 1 — Figure S1. Search strategies Figure S2. Data extraction form Figure S3. Quality assessment Data S4 Quality assessment scores Data S5. Effects of specified exposure on pregnancy outcomes in South Asian and White women [file OBR-19-485-s001.docx]

**Supplementary information**

**Effects of maternal anthropometrics on pregnancy outcomes in South Asian women: a systematic review**

Emma Slack, Institute of Health & Society, Newcastle University

Judith Rankin, Institute of Health & Society, Newcastle University

Dan Jones, Health and Social Care Institute, Teesside University

Nicola Heslehurst, Institute of Health & Society, Newcastle University

Dr Nicola Heslehurst (corresponding author) on behalf of the co-authors

Institute of Health & Society, Newcastle University, NE2 4AX, UK

Tel: +44 (0) 191 208 3823, [nicola.heslehurst@ncl.ac.uk](mailto:nicola.heslehurst@ncl.ac.uk)

# Figure S1: Search strategies

**Search strategy for Medline via OVID**

1. *Pregnancy/

2. Obstetrics/

3. Pregnan$.ti,ab.

4. Matern$.ti,ab.

5. Gravid$.ti,ab.

6. Mother.ti,ab.

7. Parent.ti,ab.

8. Or/1-7

9. Ethnic groups/

10. Culture/

11. Continental population groups/

12. (Race OR Races OR Racial OR Ethnic$ OR Intra race OR Intra Races OR Intra racial OR Intra ethnic$ OR Inter race OR Inter races OR Inter racial OR Inter ethnic$).ti,ab.

13. “Emigrants and Immigrants”/

14. Acculturation/

15. Minority groups/

16. (Asian$ OR Indian$ OR Bengali$ OR Kashmiri$ OR Gujarati$ OR Tamil$ OR Bangladesh$ OR Pakistan$ OR Sri Lanka$).ti,ab

17. (Nonwhite OR minority).ti,ab.

18. Or/9-17

19. *Obesity/ or *obesity, morbid/

20. Obes$.ti,ab.

21. *body composition/

22. *Weight gain/

23. (overweight or over weight or weight gain).ti,ab.

24. Body mass index/

25. (Bmi or body mass index).ti,ab.

26. Skinfold thickness/

27. Adiposity/ph

28. *adipose tissue/

29. Waist circumference/ph

30. Waist-hip ratio/

31. body fat percentage.mp.

32. or/19-31

33. 8 and 18 and 32

34. Fertile$.ti,ab.

35. (IVF or in vitro fertili?ation).ti.

36. (PCOS or polycystic ovary syndrome)

37. Or/34-36

38. 33 not 37

39. Limit 38 to Human

40. Limit 39

Note: .mp.=title, abstract, original title, name of substance word, subject heading word, keyword heading word, protocol supplementary concept word, rare disease supplementary concept word, unique identifier

**Search strategy for EMBASE via OVID**

1. Pregnancy/
2. Obstetrics/
3. Pregnan$.ti,ab.
4. Matern$.ti,ab.
5. Gravid$.ti,ab.
6. Mother.ti,ab.
7. Parent.ti,ab.
8. Or/1-7
9. Ethnic group/
10. Ethnicity.ti,ab
11. Race/
12. Cultural anthropology/
13. Ancestry group/
14. (Race OR Racial OR Ethnic$ OR Intra race OR Intra Races OR Intra racial OR Intra ethnic$ OR Inter race OR Inter races OR Inter racial OR Inter ethnic$).ti,ab.
15. Emigrant/
16. Migrant/
17. Cultural factor/
18. Minority group/
19. (Asian$ OR Indian$ OR Bengali$ OR Kashmiri$ OR Gujarati$ OR Tamil$ OR Bangladesh$ OR Pakistan$ OR Sri Lanka$).ti,ab
20. Nonwhite.ti,ab. OR minority.ti,ab.
21. Or/9-20
22. *Obesity/ or *morbid obesity/
23. Obes$.ti,ab.
24. *body composition/
25. *Weight gain/
26. (overweight or over weight or weight gain).ti,ab.
27. Body mass/
28. BMI or body mass index.ti,ab.
29. Skinfold thickness/
30. *adipose tissue/
31. Waist circumference/
32. Waist-hip ratio/
33. body fat distribution/
34. Body fat percentage.mp.
35. or/22-34
36. 8 and 21 and 35
37. Fertile$.ti,ab.
38. (IVF or in vitro fertili?ation).ti.
39. (PCOS or polycystic ovary syndrome)
40. Or/37-39
41. 36 not 40
42. Limit 41 to Human
43. Limit 42 to English

**Search strategy for PsychINFO via OVID**

1. *Pregnancy/
2. Exp Obstetrics/
3. Pregnan$.ti,ab.
4. Matern$.ti,ab.
5. Gravid$.ti,ab.
6. Mother.ti,ab.
7. Parent.ti,ab.
8. Or/1-7
9. exp "Racial and Ethnic Groups"/
10. ethnic identity/
11. exp "Racial and Ethnic Differences"/
12. exp “Race (Anthropological)"/
13. exp Minority Groups/
14. exp Immigration/
15. (Race OR Racial OR Ethnic$ OR Intra race OR Intra Races OR Intra racial OR Intra ethnic$ OR Inter race OR Inter races OR Inter racial OR Inter ethnic$).ti,ab.
16. (Asian$ OR Indian$ OR Bengali$ OR Kashmiri$ OR Gujarati$ OR Tamil$ OR Bangladesh$ OR Pakistan$ OR Sri Lanka$).ti,ab
17. Nonwhite.ti,ab. OR minority.ti,ab.
18. Or/ 9-17
19. *Obesity/
20. Obes$.ti,ab.
21. Weight gain/
22. Body weight/
23. exp Body Size/
24. exp Body Mass Index/
25. exp Body Weight/
26. exp Body Fat/
27. Or/ 19-26
28. 8 and 18 and 27
29. Fertile$.ti,ab.
30. (IVF or in vitro fertili?ation).ti.
31. (PCOS or polycystic ovary syndrome)
32. Or/29-31
33. 28 not 32
34. Limit 33 to Human
35. Limit 34 to English

**Search strategy for CINAHL via EbescoHost**

(MM "Pregnancy") OR (MH "Delivery, Obstetric+") OR (TI "pregnan*" OR AB "pregnan*") OR (TI “Matern*” OR AB “Matern*”) OR *(TI “Gravid*” OR AB “Gravid”) OR (TI “Mother” OR AB “Mother”) OR (TI “Parent” OR AB “Parent”)

AND

(MH "Ethnic Groups+") OR (TI “Ethnicity” OR AB “Ethnicity”) OR (MH "Race Relations+") OR (MH "Culture+") OR (TI “Race” OR AB “Race”) OR (TI “Racial” OR AB “Racial”) or (TI “Ethnic*” OR AB “Ethnic*) OR (TI “Intra race” OR AB “Intra race”) OR (TI “Intra Races” or AB “Intra races”) OR (TI “Intra Racial” OR AB “Intra racial”) OR (TI “Intra ethnic*” OR AB “Intra ethnic*”) OR (TI “Inter race” OR AB “Inter race”) OR (TI “Inter races” OR AB “Inter Races”) OR (TI “Inter Racial” OR AB “Inter Racial”) OR (TI “Inter ethnic*” OR AB “Inter ethnic”) OR (MH "Emigration and Immigration") OR (MH "Migrants") OR (MH "Acculturation") OR (MH "Minority Groups") OR (TI “Asian*” OR AB “Asian”) OR (TI “Indian*” OR AB “Indian*”) OR (TI “Bengali*” OR AB “Bangali*”) OR (TI “Kashmiri*” OR AB “Kashmiri*”) OR (TI “Gujarati*” OR AB “Gujarati*”) OR (TI “Tamil*” OR AB “Tamil*”) OR (TI “Bangladesh*” OR AB “Bangladesh*”) OR (TI “Pakistan*” OR AB “Pakistan*”) OR (TI “Sri Lanka* OR AB “Sri Lanka*”) OR (TI “Nonwhite minority” OR AB “Nonwhite minority”)

AND

(MM "Obesity") OR (MM "Obesity, Morbid") OR (TI “obes*” OR AB “obes*”) OR (MH "Body Weight Changes") OR (MH "Weight Gain") OR (TI “Overweight” OR AB “Overweight”) OR (TI “over weight” OR AB “over weight”) OR (TI “weight gain” OR AB “weight gain”) OR (MH "Body Mass Index") OR (TI “BMI” OR AB “BMI”) OR (TI “body mass index” OR AB “body mass index”) OR (MH "Skinfold Thickness") OR (MH "Adipose Tissue") OR (MH "Waist Circumference") OR (MH "Waist-Hip Ratio") OR (MH "Adipose Tissue Distribution") OR "body fat percentage"

NOT

(TI “fertile* OR AB “fertile*”) OR (TI “IVF” OR TI “In vitro fertili*ation”) OR “PCOS” or “polycystic ovary syndrome”

**Search strategy for the JBI database**

Pregnan* OR and Ethnicity or "South Asian" and Obesity OR Overweight OR "weight gain" OR weight

**Search strategy for Scopus, CRD database (DARE), PROSPERO**

Pregnancy OR Pregnant OR Maternal

AND

Ethnicity OR ethnic OR Minority OR race OR South Asian” OR Indian OR India OR Pakistani OR Pakistan OR Bangladesh OR Bangladeshi OR “Sri Lankan” OR “Sri Lanka”

AND

Obesity OR Overweight OR weight OR body mass OR Body Weight Changes OR “BMI” OR “Waist circumference” OR "Waist-Hip Ratio" or “Body Fat percentage”

**Search strategy for Cochrane database of systematic reviews**

1. Pregnan*.mp
2. Maternal.mp
3. Mother.mp
4. parent.mp
5. Gravid.mp
6. Gravida.mp
7. Or/1-6
8. Ethnicity.mp
9. ethnic.mp
10. Minority.mp
11. Culture.mp
12. Race.mp
13. racial.mp
14. South Asian.mp
15. India*.mp
16. Pakistan*.mp
17. Bangladesh*.mp
18. Sri Lanka*.mp
19. Or/8-18
20. Obesity.mp
21. Overweight.mp
22. adiposity.mp
23. weight.mp
24. body mass index.mp
25. Body Weight Changes.mp
26. BMI.mp
27. Waist circumference.mp
28. Waist-Hip Ratio.mp
29. Body Fat percentage.mp
30. Or/20-29
31. 7 and 19 and 30

**Search strategy for federated search engine Epistemonikos**

Pregnancy OR Pregnant OR Maternal or Mother OR parent OR Gravid or Gravida

AND

Ethnicity OR ethnic OR “ethnic group” OR Minority OR culture OR race OR racial OR migrant OR migrant OR “South Asian” OR Indian OR India OR Pakistani OR Pakistan OR Bangladesh OR Bangladeshi OR “Sri Lankan” OR “Sri Lanka”

AND

obesity OR Overweight OR “over weight” OR adiposity OR “adipose tissue” OR “weight gain” OR weight OR "body mass index" OR “body mass” OR "Body Weight Changes" OR “BMI” OR “Waist circumference” OR "Waist-Hip Ratio" or “Body Fat percentage”

**Search strategy for BNI (ProQuest)**

((((SU.EXACT("Pregnancy") OR SU.EXACT("1:Pregnancy ")) OR SU.EXACT.EXPLODE("Obstetrics")) OR (ti(pregnan* OR matern* OR gravid* OR mother OR parent) OR ab(pregnan* OR matern* OR gravid* OR mother OR parent))) AND ((SU.EXACT.EXPLODE("Ethnic Groups") OR SU.EXACT.EXPLODE("Culture and Religion")) OR (ti(Race OR Races OR Racial OR Ethnic* OR Intra race OR Intra Races OR Intra racial OR Intra ethnic* OR Inter race OR Inter races OR Inter racial OR Inter ethnic*) OR ab(Race OR Races OR Racial OR Ethnic* OR Intra race OR Intra Races OR Intra racial OR Intra ethnic* OR Inter race OR Inter races OR Inter racial OR Inter ethnic*)) OR (ti(Asian* OR Indian* OR Bengali* OR Kashmiri* OR Gujarati* OR Tamil* OR Bangladesh* OR Pakistan* OR Sri Lanka*) OR ab(Asian* OR Indian* OR Bengali* OR Kashmiri* OR Gujarati* OR Tamil* OR Bangladesh* OR Pakistan* OR Sri Lanka*)) OR (ti(Nonwhite OR minority or non-white) OR ab(Nonwhite OR minority or non-white))) AND ((SU.EXACT.EXPLODE("Obesity") OR SU.EXACT("Body Size")) OR (ti(obes* OR overweight OR over weight OR weight gain OR Bmi OR body mass index OR body composition OR Skinfold thickness OR Adiposity OR adipose tissue OR Waist circumference OR Waist-hip ratio OR body fat percentage) OR ab(obes* OR overweight OR over weight OR weight gain OR Bmi OR body mass index OR body composition OR Skinfold thickness OR Adiposity OR adipose tissue OR Waist circumference OR Waist-hip ratio OR body fat percentage)))) NOT (ab(Fertile* OR IVF OR in vitro fertilization OR IVF OR in vitro fertilisation OR PCOS OR polycystic ovary syndrome) OR ti(Fertile* OR IVF OR in vitro fertilization OR IVF OR in vitro fertilisation OR PCOS OR polycystic ovary syndrome))

**Search strategy for AMED (Allied and Complementary Medicine)**

1. exp pregnancy/

2. Mothers/

3. (pregnan* or matern* or gravid* or mother or parent).ti,ab.

4. exp ethnic groups/

5. "emigration and immigration"/

6. (Race or Races or Racial or Ethnic* or Intra race or Intra Races or Intra racial or Intra ethnic* or Inter race or Inter races or Inter racial or Inter ethnic*).ti,ab.

7. (Asian* or Indian* or Bengali* or Kashmiri* or Gujarati* or Tamil* or Bangladesh* or Pakistan* or Sri Lanka* or minority group*).ti,ab.

8. (Nonwhite or minority or non-white).ti,ab.

9. culture/

10. (Acculturation or culture or cultural or cultural characteristics or cross-cultural comparision or socio-cultural).mp.

11. or/1-3

12. or/4-9

13. obesity/

14. Body composition/

15. body mass index/

16. Adipose tissue/

17. (obes* or overweight or over weight or weight gain or Bmi or body mass index or body composition or Skinfold thickness or Adiposity or adipose tissue or Waist circumference or Waist-hip ratio or body fat percentage).ti,ab.

18. or/13-17

19. 11 and 12 and 18

# Figure S2: Data extraction form

**ADAPTED COCHRANE COHORT STUDY DATA EXTRACTION TEMPLATE**

| **Reviewer** |  |
| --- | --- |
| **Title of paper** |  |
| **Author and Year** |  |
| **Setting** | Location (region/city, country):  Study name or dataset: |
| **Data collection time period** (Day, Month, Year if available) |  |
| **Methodology (please check relevant box)** |  Prospective Cohort   Retrospective Cohort   Case Control   Cross sectional |

| **All ethnic groups studied** (Please use terminology from the paper) | **Subgroups included** |
| --- | --- |
|  |  |
|  |  |
|  |  |
|  |  |
|  |  |
|  |  |

| **How was ethnicity assigned? (Please check relevant box)** |  **Self-report**   **Country of birth**   **Parent’s country of birth**   **Investigator assigned**   **Medical records, unspecified**   **Unspecified**   **Other**  **If “Other” please specify……………………………………….......** |
| --- | --- |

| **Outcome** | **Definition** (give definition used to define/diagnose outcome) | **How outcome was determined:**  **measured/self-report/unclear** | **How data was collected:**  **routine medical records/prospectively collected for study/unclear** |
| --- | --- | --- | --- |
| **GDM** |  |  |  |
|  |  |  |  |
|  |  |  |  |
|  |  |  |  |
|  |  |  |  |
|  |  |  |  |
|  |  |  |  |
|  |  |  |  |
|  |  |  |  |

| **Exposure** (weight status before or during pregnancy i.e. BMI, weight, skinfold thickness, serum leptin or gestational weight gain) | **Definition** (please give units used and groups if applicable. Also include if Asian specific criteria used) | **How exposure was determined:**  **measured/self-report/unclear** | **When assessed (**Please give as much detail as possible e.g. 1^st^ antenatal appointment, or 16 weeks of pregnancy etc) | **Reference group used** |
| --- | --- | --- | --- | --- |
|  |  |  |  |  |
|  |  |  |  |  |
|  |  |  |  |  |
|  |  |  |  |  |

|  | Total group | White ethnic group  …………... | Asian ethnic group 1 …………… | Asian ethnic group 2 …………… | Asian ethnic group 3 …………… | Asian ethnic group 4 …………… |
| --- | --- | --- | --- | --- | --- | --- |
| Number Identified |  |  |  |  |  |  |
| Number Excluded |  |  |  |  |  |  |
| Final Number Included |  |  |  |  |  |  |
| All Subjects Accounted for in each ethnic group? | Yes  No  Unclear | Yes  No  Unclear | Yes  No  Unclear | Yes  No  Unclear | Yes  No  Unclear | Yes  No  Unclear |

(Note: Relevant Asian populations refer to South Asian, UK studies using the term Asian or any other Asian term which only includes women from South Asia using the definition used by NICE (immigrants and descendants from Bangladesh, Bhutan, India, Indian-Caribbean (immigrants of South Asian family origin), Maldives, Nepal, Pakistan and Sri Lanka) for example; Indo-Asian, Asian-Indian, Indian, Pakistani, Bangladeshi.

Relevant White ethnic groups are White, White European, Caucasian, those containing White British women etc)

| Inclusion criteria (e.g. gestation at weight measurement, singleton etc) |  |
| --- | --- |
| Exclusion criteria |  |

**Baseline Characteristics reported by ethnicity? Yes / No (**if no do not complete, if yes populate with the data**)**

| **Characteristic (**include all listed e.g. Maternal Age, Parity, Family history of diabetes, deprivation, etc and definition/unit of measurement  N/B: If population split by e.g.GDM please report GDM and Non GDM group)  Mean (sd) | Total group | White ethnic group  …………. | Asian ethnic group 1 …………… | Asian ethnic group 2 …………. | Asian ethnic group 3 …………. | Asian ethnic group 4 …………. | P value |
| --- | --- | --- | --- | --- | --- | --- | --- |
| e.g. Maternal age  GDM  Non GDM |  |  |  |  |  |  |  |
|  |  |  |  |  |  |  |  |
|  |  |  |  |  |  |  |  |
|  |  |  |  |  |  |  |  |
|  |  |  |  |  |  |  |  |
|  |  |  |  |  |  |  |  |

(Note: Relevant Asian populations refer to South Asian, UK studies using the term Asian or any other Asian term which only includes women from South Asia using the definition used by NICE (immigrants and descendants from Bangladesh, Bhutan, India, Indian-Caribbean (immigrants of South Asian family origin), Maldives, Nepal, Pakistan and Sri Lanka) for example; Indo-Asian, Asian-Indian, Indian, Pakistani, Bangladeshi

Relevant White ethnic groups are White, White European, Caucasian, those containing White British women etc)

**Are there any observed differences in baseline characteristics by ethnic group?**

**Data Analysis: please complete table and note ethnic group term used-if additional analysis or additional Asian ethnic group, please use table over page**

| Pregnancy outcome | Exposure (Maternal BMI, other pre-pregnancy weight status, GWG, skinfold thickness etc) | White ethnic group | | | | Unadjusted Statistical result  ……….…...  and………%  Confidence interval | Adjusted Statistical result ……………..  and……....% Confidence interval | Asian ethnic group | | | | Unadjusted Statistical result  ……….…...  and………%  Confidence interval | Adjusted Statistical result ……………..  and……....% Confidence interval |
| --- | --- | --- | --- | --- | --- | --- | --- | --- | --- | --- | --- | --- | --- |
|  |  | Mean (SD) | Number with outcome | Number without outcome | Total number |  |  | Mean (SD) | Number with outcome | Number without outcome | Total number |  |  |
| GDM |  |  |  |  |  |  |  |  |  |  |  |  |  |
|  |  |  |  |  |  |  |  |  |  |  |  |  |  |
|  |  |  |  |  |  |  |  |  |  |  |  |  |  |
|  |  |  |  |  |  |  |  |  |  |  |  |  |  |

(additional columns over page)

| Pregnancy outcome | Exposure (Maternal BMI, other pre-pregnancy weight status, GWG, skinfold thickness etc) | Ethnic group | | | | Unadjusted Statistical result  ……….…...  and………%  Confidence interval | Adjusted Statistical result ……………..  and……....% Confidence interval | Ethnic group | | | | Unadjusted Statistical result  ……….…...  and………%  Confidence interval | Adjusted Statistical result ……………..  and……....% Confidence interval |
| --- | --- | --- | --- | --- | --- | --- | --- | --- | --- | --- | --- | --- | --- |
|  |  | Mean (SD) | Number with outcome | Number without outcome | Total number |  |  | Mean (SD) | Number with outcome | Number without outcome | Total number |  |  |
| GDM |  |  |  |  |  |  |  |  |  |  |  |  |  |
|  |  |  |  |  |  |  |  |  |  |  |  |  |  |
|  |  |  |  |  |  |  |  |  |  |  |  |  |  |
|  |  |  |  |  |  |  |  |  |  |  |  |  |  |
|  |  |  |  |  |  |  |  |  |  |  |  |  |  |

| **Factors adjusted for in analyses** (Please only consider analysis presented in table(s) on previous page(s) with results relevant to this systematic review)**:** |
| --- |

| **Data Analysis methods** (Please only consider analysis presented in table(s) on previous page(s) with results relevant to this systematic review): |
| --- |

| **Any other relevant analysis not presented in table?** (e.g. graphs and figures where numerical data not presented) |
| --- |

# Figure S3: Quality assessment

**ADAPTED NEWCASTLE - OTTAWA QUALITY ASSESSMENT SCALE**

**COHORT**^1^ **STUDIES**

**Study (author and year):**

**Reviewer (initials):**

**Section 1: Selection**

1) Representativeness of the exposed cohort (exposure in this context is the maternal weight risk group used, e.g. obesity ≥30kg/m^2^ or the GWG risk group used e.g.>20lb for obese women)

a) truly representative of the average pregnant population in the community ****

(Did they report how representative the study population BMI/GWG distribution was to the general maternity population in their setting/location/region/country? If it was reported then was it comparable? Or did they include the entire population in the sample – e.g. all women delivering within a specific maternity unit etc)

b) somewhat representative of the average pregnant population in the community ****

(Did they report how representative the study population BMI/GWG distribution was to the general maternity population in their setting/location/region/country? If it was reported then was it a similar enough pattern of distribution and not skewed in comparison?)

c) selected group of users eg nurses, volunteers ****

(E.g. only first time pregnancy, only teenage pregnancy, only those with GDM, only those requiring a certain procedure during pregnancy etc)

d) no description of the derivation of the cohort ****

(Not reported or unclear)

2) Selection of the non exposed cohort (non-exposure is the maternal weight group used as reference e.g. recommended BMI (18.5-24.9kg/m^2^ or the GWG group used as reference e.g.11-20lbs for obese women)

a) drawn from the same community as the exposed cohort ****

**(**Probably this option most of the time if using a general population of pregnancies and determining exposure status based on splitting the group by BMI)

b) drawn from a different source

(E.g. different maternity unit, different specialist clinic, different time range for recruitment between exposed and non-exposed groups)

c) no description of the derivation of the non exposed cohort

(Not reported or unclear)

3). Ascertainment of exposure (maternal BMI/GWG/ other pregnancy weight measurement e.g. skinfold thickness)

a) secure record ****

(Explicitly stated that it was a measured weight used to inform BMI/GWG)

b) structured interview ****

(No structured interview method for measuring weight status exists. In our case this option could be if self-reported weight was used but it was subsequently validated by

measured weight)

c) written self report ****

(Any self-report weight not validated with measured weight)

d) no description

(Unclear or not explicitly reported how they derived the BMI measurement)

~~4) Demonstration that outcome of interest was not present at start of study~~ ^2^

~~a) yes~~ **~~~~**

~~b) no~~

**Section 2: Comparability**

1. Comparability of cohorts on the basis of the design or analysis (can select more than one answer) please only consider analysis with results relevant to this systematic review
2. study controls for a measure of socioeconomic status (IMD, Carstairs Index, maternal education, maternal income etc) ****

(This could be either excluded or adjusted for in analysis)

1. study controls for any additional factor ****

(Any other factors adjusted for in the analysis)

c) No factors controlled for ****

2) Assessment of pregnancy outcome. (in studies where there are multiple pregnancy outcomes which would have different responses if considered separately, please complete this question to reflect the majority of outcomes)

a) independent blind assessment****

(prospectively collected and measured outcome data for the purposes of the research study)

b) record linkage ****

(Outcome data retrieved from medical records that had been informed by routine measured data)

c) self report

(Any self-reported outcome data regardless of method of data collection) ****

d) no description

(not clear/not reported) ****

3) Was follow-up long enough for pregnancy outcomes to occur? (in studies where there are multiple pregnancy outcomes which would have different responses if considered separately, please complete this question to reflect the majority of outcomes)

a) Yes (or if retrospective analysis of routine medical records) ****

(For example;

-If GDM: follow up until diagnosis of GDM is made following relevant diagnostic test such as oral glucose tolerance test at 24-28 weeks gestation.

-If birth weight: follow up until measurement of weight after birth at neonatal examination.

-If gestational age at delivery: followed up until spontaneous onset of labour, or if there was early intervention of induction of labour or caesarean then this was after the gestational age specified as pregnancy outcome, or these factors accounted for in exclusion criteria or adjustments.)

b) No ****

(For example;

-If GDM: Failure to follow up until assessment of GDM status during pregnancy.

-If birth weight: failure to follow up until measurement of weight after birth at neonatal examination.

-If gestational age at delivery: early intervention of induction of labour or caesarean before the gestational age specified as pregnancy outcome which was not accounted for in the exclusion criteria or adjustments.)

4) Adequacy of follow up of cohorts or management of missing data

a) Complete follow up – all subjects accounted for or multiple imputation of missing data ****

(The total number of eligible participants/recruited participants are reported and the final number included are reported: no loss to follow up or exclusions of cases (e.g. missing data)

b) Subjects lost to follow up unlikely to introduce bias - small number lost to follow up <20% (select an adequate %), or description provided of those lost i.e comparison of characteristics of included participants and those with missing data ****

(The total number of eligible participants /recruited participants are reported and the final number included are reported and either: lost or excluded less than 20% so presumed unlikely to introduce bias, or lost or excluded more than 20% but compared groups and no systematic differences so presumed missing at random)

c) follow up rate < 80% (select an adequate %) and no description of those lost ****

(The total number of eligible participants/recruited participants are reported and the final number included are reported: excluded or lost more than 20% but no comparison of included or excluded groups reported)

d) No statement ****

(The total number of eligible participants/recruited participants are not reported and only the final number included are reported. No mention of any exclusions or loss to follow up)

Total number of stars (out of a possible 8^3^):

Notes:

^1^ All the non-cohort studies were cross sectional and all had groups defined by the exposure variable rather than the outcome variables, therefore cohort design template fits best with these study

^2^ Item 4 in Section 1: Selection “Demonstration that outcome of interest was not present at start of study” is not applicable to gestational age at delivery outcomes as women are identified in early pregnancy using their pre/early pregnancy BMI and their pregnancy outcomes are not known at the start of the study. Therefore this item has been removed from the scale

^3^ A study can be awarded a maximum of one star for each numbered item within the Selection and Outcome categories. A maximum of two stars can be given for Comparability. The denominator value for the possible number of stars using the template Newcastle Ottawa Scale has been reduced from 9 to 8 due to the removal of item 4 in Section 1 (as there was potential for additional star to be awarded based on this item).

# Supplementary information 4 Quality assessment scores

| **Paper** | Section 1:Selection | | | Section 2: Comparability | | | | Final score (Max:8) | Reviewers |
| --- | --- | --- | --- | --- | --- | --- | --- | --- | --- |
|  | 1 | 2 | 3 | 1 | 2 | 3 | 4 |  |  |
| Anand et al 2016^45^ | A* | A* | C | B* | A* | A* | D | 5 | ES + DJ |
| Bissenden et al 1981^31^ | D | A* | D | C | D | A* | D | 2 | ES+JR |
| Bissenden et al 1981^30^ | D | A* | D | C | D | A* | D | 2 | ES+NH |
| Bryant et al 2014^28^ | A* | A* | A* | C | B* | A* | C | 5 | ES + DJ |
| Davies-Tuck et al 2016^48^ | A* | A* | C | A+B** | B* | A* | D | 7 | ES+DJ |
| Dornhost et al 1992^35^ | A* | A* | D | C | A* | A* | A* | 5 | ES+JR |
| Dunne et al 2000^38^ | C | A* | D | C | B* | A* | D | 3 | ES+DJ |
| Hernandez-Rivas et al 2013^47^ | C | A* | D | C | A* | A* | B* | 4 | ES+DJ |
| Makgoba et al 2011^33^ | A* | A* | C | C | A* | A* | B* | 5 | ES+DJ |
| Makgoba et al 2012^34^ | C | A* | C | A+B** | B* | A* | C | 5 | ES+NH |
| Nishikawa et al 2017^39^ | A* | A* | A* | A+B** | B* | A* | D | 7 | ES+DJ |
| Oteng-Ntim et al 2013^32^ | A* | A* | D | A+B** | B* | A* | B* | 7 | ES+DJ |
| Penn et al 2014^29^ | A* | A* | D | B* | B* | A* | A* | 6 | ES+DJ |
| Pu et al 2015^46^ | A* | A* | D | A+B** | B* | A* | B* | 7 | ES +DJ |
| Retnakaran et al 2005^44^ | C | A* | D | C | A* | A* | D | 3 | ES+DJ |
| Sharma et al 2011^36^ | C | A* | D | C | A* | A* | B* | 4 | ES+DJ |
| Sheridan et al 2013^27^ | C | A* | B* | C | B* | A* | B* | 5 | ES+DJ |
| Sinha et al 2002^37^ | C | A* | D | B* | B* | A* | C | 4 | ES+DJ |
| Sommer et al 2015^43^ | C | A* | A* | B* | A* | A* | C | 5 | ES+DJ |
| Sommer et al 2014^42^ | C | A* | A* | B* | A* | A* | B* | 6 | ES+NH |
| Wong et al 2011^40^ | C | A* | D | C | B* | A* | B* | 4 | ES+DJ |
| Yue et al 1996^41^ | A* | A* | D | C | A* | A* | D | 4 | ES+JR |

**Supplementary information 5** Effects of specified exposure on pregnancy outcomes in South Asian and White women

| **Author and study year** | **Ethnic groups** | **Exposure** | **Control group** | **Pregnancy outcome** | **OR (95%CI)** | | **AOR (95%CI)** | |
| --- | --- | --- | --- | --- | --- | --- | --- | --- |
|  |  |  |  |  | **White ethnic group** | **South Asian ethnic group** | **White ethnic group** | **South Asian ethnic group** |
| Bryant et al 2014^28^ | White British women (n=4547) | 5kg/m^2^ increase in BMI | n/a | GDM | 1.25 (1.12, 1.40)* | 1.55 (1.43, 1.69)* | - | - |
|  |  |  |  | Preterm birth | 0.87 (0.77, 0.98)* | 0.98 (0.87, 1.11) | - | - |
|  | Pakistani women (n=4547) |  |  | Macrosomia | 1.36 (1.27, 1.47)* | 1.57 (1.41, 1.75)* | - | - |
|  |  |  |  | Hypertensive disorder | 1.60 (1.46, 1.76)* | 1.54 (1.39, 1.71)* | - | - |
|  |  |  |  | C-Section | 1.34 (1.26, 1.42)* | 1.36 (1.27, 1.45)* | - | - |
| Davies-Tuck et al 2016^48^ | White women; Australian and New Zealand (n=18768)  South Asian women; women from Afghanistan, Bangladesh, Bhutan. India, Iran, Maldives, Nepal, Pakistan and Sri Lanka (n=8342) | ≥30kg/m^2^ | <30kg/m^2^ | Hypertension | 1.98 (1.76, 2.24) ^∞*^ | 1.47 (1.07, 2.04) ^∞*^ | 3.14 (2.78, 3.54)*^a^ | 1.59 (1.14, 2.21)*^a^ |
|  |  |  |  | GDM | 3.20 (2.80, 3.65) ^∞*^ | 3.48 (2.88, 4.21) ^∞*^ | 3.21 (2.80, 3.67)*^b^ | 1.89 (1.57, 2.28)*^b^ |
|  |  |  |  | Preterm birth | 1.06 (0 .95, 1.18)^∞^ | 1.07 (0.80, 1.43)^∞^ | 1.04 (0.93, 1.16)^c^ | 1.08 (0.81, 1.45)^c^ |
|  |  |  |  | Shoulder dystocia | 1.18 (0.93,1.50) ^∞^ | 2.15 (1.43, 3.25) ^∞*^ | 1.11 (0.87, 1.41)^d^ | 1.99 (1.26, 2.96)*^d^ |
|  |  |  |  | PPH | 1.52 (1.33, 1.74)^∞*^ | 1.27(0.93, 1.7) ^∞*^ | 1.49 (1.30, 1.71)*^e^ | 1.32 (0.96, 1.82)^e^ |
|  |  |  |  | Induced labour | 1.48 (1.37, 1.58)^∞*^ | 1.29 (1.10, 1.50)^∞*^ | - | - |
|  |  |  |  | Instrumental vaginal | 0.65 (0.59, 0.72)^∞*^ | 0.60 (0.48, 0.74)^∞*^ | 0.73 (0.65, 0.82)*^f^ | 0.76 (0.57, 1.01)^f^ |
|  |  |  |  | Unplanned C-section | 1.49 (1.37, 1.62)^∞*^ | 1.37 (1.16, 1.62)^∞*^ | 1.35 (1.23, 1.49)*^g^ | 1.38 (1.15, 1.66)*^g^ |
|  |  |  |  | SGA | 0.64 (0.57, 0.72)^∞*^ | (0.56 (0.45, 0.71)^∞*^ | 0.64 (0.57, 0.72)*^h^ | 0.64 (0.51, 0.81)*^h^ |
|  |  |  |  | Macrosomia | 1.43 (1.31, 1.56)^∞*^ | 2.22 (1.78, 2.77) ^∞*^ | 1.90 (1.73, 2.08)*^i^ | 2.24 (1.75, 2.83*^i^ |
|  |  |  |  | Foetal compromise | 1.08 (1.00, 1.16)^∞*^ | 1.03 (0.88, 1.19)^∞^ | 1.19 (1.02, 1.28)*^j^ | 1.31 (1.12, 1.53)*^j^ |
|  |  |  |  | Admission to NICU/SCN | 1.30 (1.20, 1.40)^∞*^ | 1.52 (1.29, 1.78)^∞*^ | 1.33 (1.22, 1.45)*^k^ | 1.66 (1.40, 1.97)*^k^ |
|  |  |  |  | Any perinatal morbidity | 1.14 (1.07, 1.22)^∞*^ | 1.34 (1.17, 1.54)^∞*^ | 1.18 (1.04, 1.27)*^l^ | 1.45 (1.25, 1.68)*^l^ |
|  |  |  |  | Stillbirth | 1.14 (0.75, 1.73)^∞^ | 1.60 (0.69, 3.75)^∞^ | 0.90 (0.57, 1.42)^m^ | 1.42 (0.55, 3.63)^m^ |
| Dornhorst et al 1992^35^ | White women; Northern European and Caucasian (n=6109)  Indian women; from the Indian subcontinent (n=1164) | BMI ≥27 kg/m^2^ | BMI <27 kg/m^2^ | GDM | 4.6 (2.1,10.4)* | 3.5 (2.0, 4.2)* | 4.3 (1.9, 9.8)*^n^ | 2.0 (0.9, 4.2)^n^ |
| Makgoba et al 2011^33^ | White woman (n=131201) | 25.0-29.9 kg/m^2^ | 15.5-24.9kg/m^2^ | GDM | 1.77 (1.50, 2.09)^*^ | 2.57 (2.02, 3.23) ^∞*^ | - | - |
|  | South Asian women (n=2749) | ≥30kg/m^2^ |  |  | 4.70 (3.98, 5.55)^*^ | 5.80 (4.36, 7.71) ^∞*^ | - | - |
| **Author and study year** | **Ethnic groups** | **Exposure** | **Control group** | **Pregnancy outcome** | **OR (95%CI)** | | **AOR (95%CI)** | |
|  |  |  |  |  | **White ethnic group** | **South Asian ethnic group** | **White ethnic group** | **South Asian ethnic group** |
| Oteng-Ntim 2013^32^ | White women; White British, White Irish and Other White (n=12418)  Asian women; Bangladeshi, Indian, Pakistani, other Asian and Asian British (n=1162) | ≥30kg/m^2^ | <30kg/m^2^ | Diabetes (GDM and pre-existing diabetes) | - | - | 4.97 (3.39, 7.28)*^o^  **PAF** 20.3 (15.46, 24.53) | 5.48 (2.43, 12.35)*^o^  **PAF** 17.37 (13.07, 21.09) |
|  |  |  |  | Elective LSCS | - | - | 1.41 (1.08, 1.84)*^o^  **PAF** 4.24 (2.43, 6.00) | 1.52 (0.73, 3.14)^o^  **PAF** 4.02 (2.31, 5.70) |
|  |  |  |  | Emergency LSCS | - | - | 1.98 (1.69, 2.33)*^o^  **PAF** 3.48 (2.65, 4.30) | 0.65 (0.32, 1.31)^o^  **PAF** 2.93 (2.23, 3.63) |
|  |  |  |  | Instrumental Delivery | - | - | 0.78 (0.63, 0.96)*^o^  **PAF** -1.84 (-2.71, -0.98) | 1.04 (0.50, 2.16)^o^  **PAF** -1.57 (-2.30, -0.84) |
|  |  |  |  | PPH | - | - | 1.75 (1.49, 2.06)*^o^  **PAF** 3.55 (2.67, 4.41) | 0.77 (0.40, 1.48)^o^  **PAF** 3.28 (2.47, 4.09) |
|  |  |  |  | Preterm delivery | - | - | 1.66 (1.30, 2.11)*^o^  **PAF** 2.66 (1.06, 4.23) | 1.25 (0.61, 2.56)^o^  **PAF** 2.39 (0.96, 3.81) |
|  |  |  |  | Macrosomia | - | - | 1.54 (1.27, 1.89)*^o^  **PAF** 5.15 (3.64, 6.64) | 0.98 (0.30, 3.20)^o^  **PAF** 5.52 (3.84, 7.18) |
|  |  |  |  | LBW | - | - | 0.75 (0.58, 0.98)*^o^  **PAF** -0.01 (-0.10, 0.08) | 0.92 (0.47, 1.37)^o^  **PAF** -0.03 (-0.20, 0.14) |
|  |  |  |  | Admission to NICU | - | - | 1.92 (1.52, 1.42)*^o^  **PAF** 3.75 (2.05, 5.41) | 1.12 (0.52, 2.42)^o^  **PAF** 3.52 (1.94, 5.07) |
|  |  |  |  | Perinatal death | - | - | 2.19 (0.96, 4.98)^o^  **PAF** 3.17 (-2.96, 8.93) | 2.00 (0.46, 8.71)^o^  **PAF** 3.02 (-2.78, 8.50) |
| **Author and study year** | **Ethnic groups** | **Exposure** | **Control group** | **Pregnancy outcome** | **OR (95%CI)** | | **AOR (95%CI)** | |
|  |  |  |  |  | **White ethnic group** | **South Asian ethnic group** | **White ethnic group** | **South Asian ethnic group** |
| Penn et al 2014^29^ | White women; British, Irish, White Other (n=26390)  Asian women; Indian, Pakistani, Bangladeshi, Asian Other (n=2857) | ≥30kg/m^2^ | <30kg/m^2^ | Stillbirth | 1.38 (0.72, 2.66)^∞^ | 4.84 (1.97, 11.91)^∞*^ | 1.32 (0.68, 2.57)^p^ | 4.64 (1.84, 11.70)*^p^ |
|  |  | ≥27.5kg/m^2^ | <27.5kg/m^2^ |  |  |  |  | 2.83 (1.17, 6.85)*^p^ |
| Pu et al 2015^46^ | Non-Hispanic White (n=9011) | ≥25kg/m^2^ | <25kg/m^2^ | GDM | - | - | 2.0 (1.74, 2.4)*^$q^  **PAF** 28.9 (22.4, 35.1) | 1.17 (1.5, 2.0)* ^$q^  **PAF** 25.5 (17.4, 33.3)  1.9 (1.7, 2.2)* ^$q^  **PAF** 39.0 (29.7, 47.6) |
|  | Asian Indian women (n=5069) | ≥23kg/m^2^ | <23kg/m^2^ |  | - | - | - |  |
| Sheridan et al 2013^27^ | White British  (n=4488) | <18.5kg/m^2^ | 18.5-24.9kg/m^2^ | Congenital anomalies | 1.50 (0.47-4.18)^$^ | 0.96 (0.54,1.73)^$^ | - | - |
|  | Pakistani (n=5127) | 25-29.9 kg/m^2^ |  |  | 1.00 (0.59,1.70)^$^ | 1.03 (0.76,1.39)^$^ | - | - |
|  |  | ≥30kg/m^2^ |  |  | 1.22 (0.73, 2.04)^$^ | 0.69 (0.45,1.03)^$^ | - | - |
| **Author and study year** | **Ethnic groups** | **Exposure** | **Control group** | **Pregnancy outcome** | **OR (95%CI)** | | **AOR (95%CI)** | |
|  |  |  |  |  | **White ethnic group** | **South Asian ethnic group** | **White ethnic group** | **South Asian ethnic group** |
| Sommer et al 2014^42^ | European women (n=348)  South Asian women (n=181) | Weight gain (kg per week) | European women | GDM | - | - | 1 | Model 1^r^ 2.43 (1.62, 3.65)  Model 2^s^ 2.77 (1.83, 4.21)  Model 3^t^ 1.84 (1.16, 2.90) |
|  |  | Fat mass gain (kg per week) |  |  | - | - | 1 | Model 1^r^ 2.46 (1.64, 3.69)  Model 2^s^ 2.80 (1.84, 4.26)  Model 3^t^ 1.86 (1.18, 2.95) |
|  |  | Truncal fat gain (kg) |  |  | - | - | 1 | Model 1^r^ 2.44 (1.62, 3.65)  Model 2^s^ 2.78 (1.83, 4.22)  Model 3^t^ 1.82 (1.15, 2.89) |
|  |  | Mean skinfold gain (mm) |  |  | - | - | 1 | Model 1^r^ 2.50 (1.62, 3.84)  Model 2^s^ 2.72 (1.75, 4.23)  Model 3^t^ 1.88 (1.16, 3.04) |

OR=odds ratio AOR= adjusted odds ratio, GDM=gestational diabetes, NICU= neonatal intensive care unit, SCN= special care nursery, PPH=postpartum haemorrhage, SGA=small for gestational age, LSCS=lower section caesarean section, LBW=low birth weight

^∞^Effect size calculated from data provided in published paper using STATA 14

*Significant as 95% confidence interval does not cross 1.00

^$^Relative risk

PAF: population attributable fraction % and 95%CI (PAF is the reduction in population disease risk or mortality that would occur if the exposure to a risk factor was eliminated or reduced to an ideal exposure scenario, where the distributions of other risk factors in the population remain unchanged^51, 52^

^a^ adjusted for age, parity and smoking status

^b^ adjusted for age, parity and smoking status

^c^ adjusted for age, parity and smoking

^d^ adjusted for age, parity, induction, augmentation and epidural

^e^ age, parity, placental abnormality, baby birth weight, gestational hypertension, onset of labour, birth type (e.g. vaginal/instrumental/operative), episiotomy, Length of labour and pre-existing maternal blood disorder

^f^ adjusted for maternal age, parity, onset of birth, epidural, baby birth weight, gestation, head position, augmentation and account class

^g^ adjusted for maternal age, parity, account class, previous caesarean, onset of labour, gestation, birth weight, augmentation, epidural

^h^ adjusted for maternal age, parity, smoking and account class

^i^ adjusted for parity, maternal age, account class, smoking, gestation and baby gender

^j^ adjusted for maternal age, parity, smoking, gestation and baby gender

^k^ adjusted for parity, account class, GDM, gestation, baby gender, onset of labour, birth type

^l^ adjusted for maternal age, parity, account class, gestation, baby gender, onset of labour, birth type

^m^ adjusted for maternal age, parity, previous caesarean, account class, baby gender, gestation and smoking

^n^ adjusted for age and parity

^o^ adjusted for age parity and deprivation

^p^ adjusted for maternal age, hypertension and parity

^q^ adjusting for maternal education, parity, smoking and insurance status

^r^ adjusting for ethnic origin, gestational week at inclusion, age and parity

^s^ adjusting for ethnic origin, gestational week at inclusion, age, parity and pre-pregnancy BMI

^t^ adjusting for ethnic origin, gestational week at inclusion, age, parity, pre-pregnancy BMI and HOMA-IR
